# Tocilizumab versus sarilumab among adults hospitalised with COVID-19: target trial emulation across England and Scotland

**DOI:** 10.1038/s41467-026-73134-9

**Published:** 2026-05-15

**Authors:** Bang Zheng, Amanj Kurdi, Alain Amstutz, Amelia Green, Emily Herrett, John Tazare, Qing Wen, Viyaasan Mahalingasivam, Rebecca Smith, Brian MacKenna, Amir Mehrkar, Seb Bacon, Ben Goldacre, Chris Robertson, Aziz Sheikh, Laurie Tomlinson

**Affiliations:** 1 Department of Epidemiology & Biostatistics, School of Public Health, Peking University, Beijing, China; 2 Key Laboratory of Epidemiology of Major Diseases (Peking University), Ministry of Education, Beijing, China; 3 Department of Non-communicable Disease Epidemiology, London School of Hygiene and Tropical Medicine, London, UK; 4 Peking University Center for Public Health and Epidemic Preparedness & Response, Beijing, China; 5 Strathclyde Institute of Pharmacy and Biomedical Sciences, University of Strathclyde, Glasgow, UK; 6 Public Health Scotland, Edinburgh, UK; 7 College of Pharmacy, Hawler Medical University, Erbil, Iraq; 8 Al-Kitab University, Kirkuk, Iraq; 9 Department of Public Health Pharmacy and Management, School of Pharmacy, Sefako Makgatho Health Sciences University, Pretoria, South Africa; 10 Electronic Health Records Group, Population Health Sciences, Bristol Medical School, University of Bristol, Bristol, UK; 11 Oslo Center for Biostatistics and Epidemiology, Oslo University Hospital, Oslo, Norway; 12 Division Clinical Epidemiology, Department of Clinical Research, University Hospital Basel and University of Basel, Basel, Switzerland; 13 Nuffield Department of Primary Care Health Sciences, University of Oxford, Oxford, UK; 14 Department of Medical Statistics, London School of Hygiene and Tropical Medicine, London, UK; 15 Centre for Public Health, School of Medicine, Dentistry and Biomedical Sciences, Queen’s University Belfast, Belfast, UK; 16 Department of Mathematics and Statistics, University of Strathclyde, Glasgow, UK; 17 Usher Institute, University of Edinburgh, Edinburgh, UK

**Keywords:** Viral infection, Therapeutics, Respiratory signs and symptoms

## Abstract

The interleukin-6 (IL-6) inhibitors tocilizumab and sarilumab have been repurposed for COVID-19 treatment. However, discrepancies exist across global and national COVID-19 guidelines, with limited data on the comparative effectiveness between these therapeutics especially during the delta/omicron periods. With the approval of NHS England and Public Health Scotland, we compared their effectiveness among adults hospitalised with COVID-19 using electronic health records data through the OpenSAFELY-TPP (England) and EAVE II (Scotland) platforms. Following the target trial emulation framework, 10,487 patients treated between July 2021 and February 2022, when both drugs were frequently prescribed, were included. In England, 1150 (20.1%) of 5710 participants receiving tocilizumab died by day 28 compared with 820 (20.4%) of 4025 participants receiving sarilumab (adjusted hazard ratio [aHR] 1.07, 95% CI 0.96-1.19). In Scotland, 114 (29.4%) of 388 participants receiving tocilizumab died by day 28 compared with 97 (27.0%) of 359 participants receiving sarilumab (aHR 0.92, 95% CI 0.68-1.23). There was no evidence of a difference in time to hospital discharge between the groups, and no credible effect modification by variant of concern, vaccination status, age, sex, ethnicity, body mass index, or comorbidities. Our findings provide supportive evidence for both drugs as alternative therapeutic options in COVID-19 in-patient management.

## Abstract (web summary)

Using electronic health record databases in England and Scotland, this study compared tocilizumab and sarilumab in over 10,000 hospitalised COVID-19 patients and found no meaningful difference in survival, supporting both as acceptable treatments.

## Introduction

During the COVID-19 pandemic, several potential drug repurposing candidates were investigated for their effect in managing severe cases of COVID-19 infection, including interleukin-6 (IL-6) inhibitors^1^. Traditionally used for the treatment of rheumatoid arthritis, IL-6 inhibitors block IL-6 receptors that play a pivotal role in inflammatory processes^2^ and therefore represent a promising treatment option for severe COVID-19 when the inflammatory phase dominates the pathogenesis^3^. Tocilizumab and sarilumab were the most frequently evaluated IL-6 inhibitors in randomised trials^4^.

Several meta-analyses have pooled the evidence from randomised trials assessing the benefit and harm of IL-6 inhibitors among patients hospitalised with COVID-19^1,4–7^. These studies concluded that the evidence for tocilizumab in preventing death and improving clinical outcomes is moderate to high, while the evidence for sarilumab has been graded as low. The recently published Randomised, Embedded, Multi-factorial Adaptive Platform Trial for Community-Acquired Pneumonia (REMAP-CAP) trial provided evidence that the two IL-6 inhibitors have equivalent effectiveness at improving survival and reducing duration of organ support^8^. However—except for a small single-centre trial that provided some evidence on the efficacy of tocilizumab among hospitalised patients with COVID-19 pneumonia^9^—all these trials were conducted before the delta/omicron SARS-CoV-2 variants of concerns and their sub-lineages became globally prevalent and before large-scale vaccination programmes were in place.

In the United Kingdom, both tocilizumab and sarilumab were added to the National Health Service (NHS) and the National Institute for Health and Care Excellence (NICE) Rapid Guideline on managing COVID-19 in January 2021^10,11^. Initially, NHS and NICE recommended sarilumab when tocilizumab was unavailable or contraindicated, but in March 2023, they stopped recommending sarilumab entirely due to its off-label status^12^. The World Health Organization (WHO) living guideline, on the other hand, has and continues to recommend both tocilizumab and sarilumab as equal treatment options^5^.

Despite the global pandemic being over, COVID-19 still leads to hospitalisations among high-risk patient populations, highlighting the ongoing clinical needs. However, no randomised controlled trial compared sarilumab versus tocilizumab during the delta/omicron period, and no large-scale comparative effectiveness study of the two IL-6 inhibitors on COVID-19 outcomes has been conducted. To inform clinical guidelines and provide evidence for this knowledge gap, we conducted a target trial emulation study to compare the effectiveness of tocilizumab and sarilumab among adults hospitalised due to COVID-19 across England and Scotland between July 2021 and February 2022, when delta/omicron were prevalent and both drugs were frequently prescribed^13^.

## Results

We identified 9740 eligible COVID-19 patients in England (Table 1) and 747 in Scotland (Table 2) treated with an IL-6 inhibitor between July 1, 2021 and February 28, 2022. Of these, 4025 (41.3%) in England and 359 (48.1%) in Scotland received sarilumab. Those prescribed sarilumab in England were more likely to be of White ethnicity (84% sarilumab vs 79% tocilizumab), which was not the case in Scotland. Participants in the tocilizumab group were more recently vaccinated (median time from last vaccination to baseline of 176 days among sarilumab versus 128 days among tocilizumab in England and 167 days versus 140 days in Scotland). There were geographic prescription variations in England, and greater use of sarilumab during the omicron period, i.e. later in the study period across both nations.

**Table 1:** Baseline characteristics in OpenSAFELY—England

Characteristic	Levels	Tocilizumab	Sarilumab	Total
Number of patients		5710 (100)	4025 (100)	9740 (100)
Age, in years, mean (SD)		58.1 (17.1)	59.4 (15.8)	58.6 (16.6)
Age groups	18–39	955 (16.7)	480 (11.9)	1430 (14.7)
	40–59	2005 (35.1)	1530 (38)	3540 (36.3)
	≥60	2750 (48.2)	2015 (50.1)	4765 (48.9)
Sex	Male	3610 (63.2)	2465 (61.2)	6075 (62.4)
	Female	2105 (36.9)	1560 (38.8)	3665 (37.6)
Ethnicity	Black	335 (5.9)	175 (4.3)	510 (5.2)
	Mixed	120 (2.1)	70 (1.7)	190 (2)
	Other	170 (3)	100 (2.5)	265 (2.7)
	Asian	495 (8.7)	265 (6.6)	765 (7.9)
	White	4525 (79.2)	3375 (83.9)	7895 (81.1)
	Missing	70 (1.2)	45 (1.1)	115 (1.2)
Index of Multiple Deprivation	1—Most deprived	1575 (27.6)	1110 (27.6)	2685 (27.6)
	2	1240 (21.7)	875 (21.7)	2115 (21.7)
	3	1075 (18.8)	770 (19.1)	1845 (18.9)
	4	915 (16)	635 (15.8)	1550 (15.9)
	5—Least deprived	730 (12.8)	505 (12.5)	1235 (12.7)
	Missing	175 (3.1)	130 (3.2)	305 (3.1)
Region NHS	East of England	1080 (18.9)	625 (15.5)	1700 (17.5)
	London	560 (9.8)	280 (7)	840 (8.6)
	Midlands	1535 (26.9)	925 (23)	2460 (25.3)
	North East and Yorkshire	1205 (21.1)	1265 (31.4)	2470 (25.4)
	North West	255 (4.5)	150 (3.7)	405 (4.2)
	South East	285 (5)	255 (6.3)	540 (5.5)
	South West	795 (13.9)	535 (13.3)	1330 (13.7)
Rural/Urban	Urban major conurbation	1170 (20.5)	835 (20.7)	2005 (20.6)
	Urban minor conurbation	405 (7.1)	290 (7.2)	695 (7.1)
	Urban city and town	3020 (52.9)	2115 (52.5)	5135 (52.7)
	Rural town and fringe	565 (9.9)	395 (9.8)	960 (9.9)
	Rural village and dispersed	375 (6.6)	260 (6.5)	635 (6.5)
	Missing	175 (3.1)	130 (3.2)	310 (3.2)
Days since hospital admission^a^, median (IQR)		1 (0–2)	1 (0–2)	1 (0–2)
Days since 01.07.2021 (calendar period), median (IQR)		72 (38–143)	132 (110–158)	113 (58–153)
Body mass index	Underweight/normal	705 (12.3)	465 (11.6)	1170 (12)
	Overweight	1375 (24.1)	965 (24)	2340 (24)
	Obese	1280 (22.4)	890 (22.1)	2170 (22.3)
	Severely obese	1325 (23.2)	1065 (26.5)	2390 (24.5)
	Missing	1030 (18)	645 (16)	1675 (17.2)
Chronic kidney disease stages 3–5		780 (13.7)	560 (13.9)	1335 (13.7)
Severe liver disease		65 (1.1)	40 (1)	105 (1.1)
Diabetes mellitus		1755 (30.7)	1370 (34)	3130 (32.1)
Chronic cardiac disease		980 (17.2)	690 (17.1)	1670 (17.1)
Arterial hypertension		2405 (42.1)	1765 (43.9)	4175 (42.9)
Chronic respiratory disease		1435 (25.1)	1060 (26.3)	2495 (25.6)
Solid cancer		840 (14.7)	540 (13.4)	1380 (14.2)
Haematological diseases		290 (5.1)	150 (3.7)	440 (4.5)
Immunosuppressive treatment		400 (7)	305 (7.6)	705 (7.2)
Immunosuppressive disease		30 (0.5)	25 (0.6)	55 (0.6)
Solid organ transplant		115 (2)	65 (1.6)	180 (1.8)
Days since positive test^b^, median (IQR)		1 (0–3)	1 (0–3)	1 (0–3)
Days since vaccination, median (IQR)		128 (85–175)	176 (123–198)	150 (93–190)
Vaccination status	Un-vaccinated	3025 (53)	1940 (48.2)	4960 (50.9)
	One vaccination	225 (3.9)	90 (2.2)	315 (3.2)
	Two vaccinations	1970 (34.5)	1600 (39.8)	3570 (36.7)
	Three or more vaccinations	495 (8.7)	395 (9.8)	890 (9.1)
Treated during Omicron (after 06.12.2021)		1210 (21.2)	1030 (25.6)	2240 (23)
History of COVID-19 treatment		660 (11.6)	415 (10.3)	1080 (11.1)
COVID-19 re-infection (COVID-19 event ≥3 months)		80 (1.4)	55 (1.4)	130 (1.3)

*SD* standard deviation, *IQR* interquartile range.

^a^Missing: 130 (2.3%) in tocilizumab, 80 (2%) in sarilumab.

^b^Missing: 365 (6.4%) in tocilizumab, 240 (6.0%) in sarilumab.

**Table 2:** Baseline characteristics in EAVE II—Scotland

Characteristic		Levels		Tocilizumab	Sarilumab	Total
Number of patients				388 (100)	359 (100)	747 (100)
Age, in years, mean (SD)				60.9 (15)	60.1 (14.5)	60.5 (14.8)
Age groups		18–39		40 (10.3)	38 (10.6)	78 (10.4)
		40–59		131 (33.8)	135 (37.6)	266 (35.6)
		≥60		217 (55.9)	186 (51.8)	403 (53.9)
Sex		Female		145 (37.4)	136 (37.9)	281 (37.6)
		Male		243 (62.6)	223 (62.1)	466 (62.4)
Ethnicity		White		155 (39.9)	137 (38.2)	292 (39.1)
		Other		3 (0.8)	7 (1.9)	10 (1.3)
		Unknown		230 (59.3)	215 (59.9)	445 (59.6)
Index of Multiple Deprivation		1—Most deprived		138 (35.6)	163 (45.4)	301 (40.3)
		2		98 (25.3)	77 (21.4)	175 (23.4)
		3		71 (18.3)	50 (13.9)	121 (16.2)
		4		43 (11.1)	34 (9.5)	77 (10.3)
		5—Least deprived		38 (9.8)	35 (9.7)	73 (9.8)
Days since hospital admission, median (IQR)				1 (0–2)	1 (0–2)	1 (0–2)
Days since 01.07.2021, median (IQR)				75 (54–104)	139 (115–173)	109 (73–154)
Body mass index		Underweight and normal		27 (7)	22 (6.1)	49 (6.6)
		Overweight		64 (16.5)	58 (16.2)	122 (16.3)
		Obese		150 (38.7)	152 (42.3)	302 (40.4)
		Missing		147 (37.9)	127 (35.4)	274 (36.7)
Chronic kidney disease stages 3–5				54 (13.9)	41 (11.4)	95 (12.7)
Severe liver disease				8 (2.1)	7 (1.9)	15 (2)
Diabetes mellitus				99 (25.5)	79 (22)	178 (23.8)
Chronic cardiac disease				109 (28.1)	91 (25.3)	200 (26.8)
Arterial hypertension				77 (22.3)	50 (14.9)	127 (18.6)
Chronic respiratory disease				104 (26.8)	103 (28.7)	207 (27.7)
Haematological diseases				17 (4.4)	14 (3.9)	31 (4.1)
Days since vaccination, median (IQR)				140 (100–168)	167 (98–198)	150 (99–184)
Vaccination status		Un-vaccinated		134 (34.5)	146 (40.7)	280 (37.5)
		One vaccination		124 (32)	104 (29)	228 (30.5)
		Two or more vaccinations		130 (33.5)	109 (30.4)	239 (32)
Treated during omicron (after 06.12.2021)				47 (12.1)	132 (36.8)	179 (24)

*SD* standard deviation, *IQR* interquartile range.

**Fig. 1:**
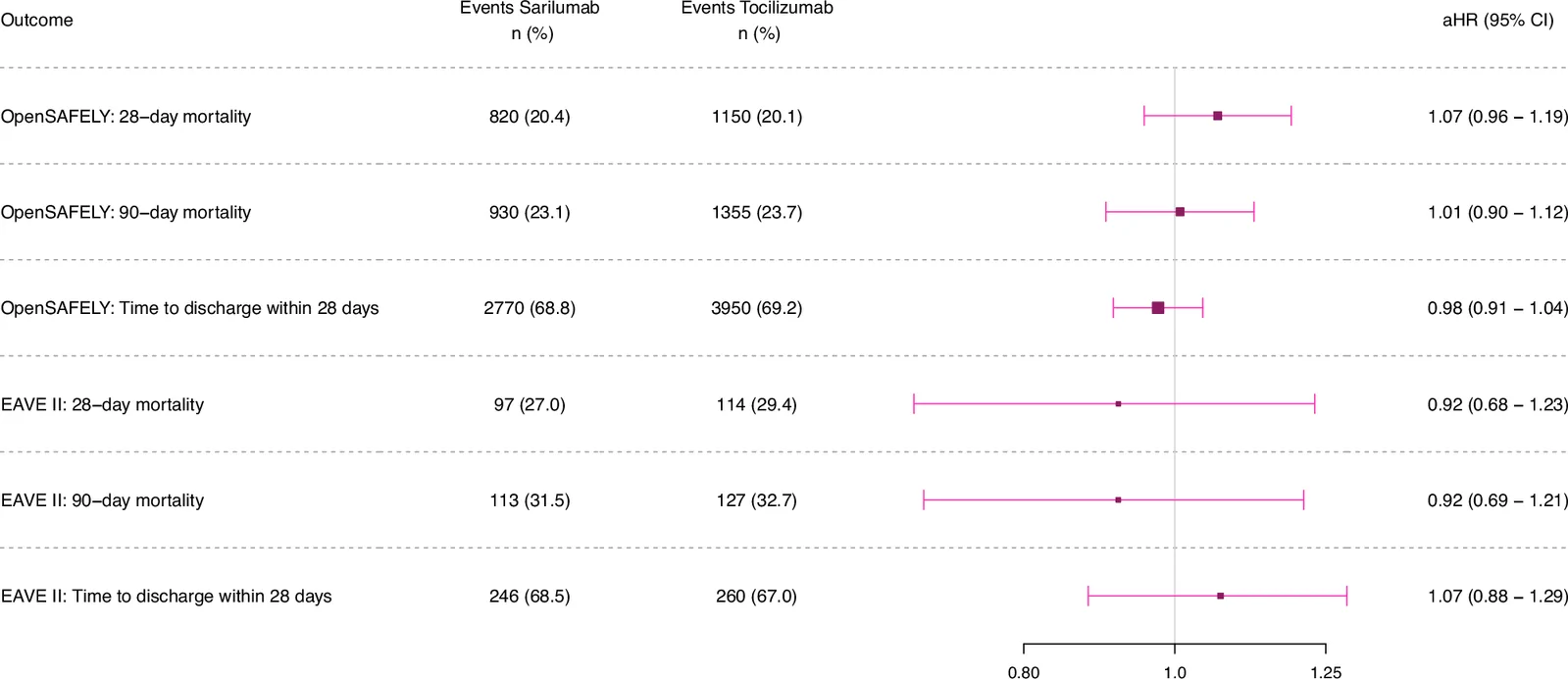
Primary and secondary outcomes. The square represents the point estimate of the adjusted hazard ratio for each outcome, with its size proportional to the estimation precision; the error bars represent the corresponding 95% confidence interval. Summary-level source data are provided as a Source data file. aHR adjusted hazard ratio, CI confidence interval.

We emulated a pragmatic target trial on the comparative effectiveness separately across the two nations. The details of the study design are presented in the Supplementary Table 1. In England, 820 (20.4%) of 4025 participants in the sarilumab group died by day 28 compared with 1150 (20.1%) of 5710 participants in the tocilizumab group. After controlling for age, sex, NHS region, calendar time, ethnicity, Index of Multiple Deprivation, COVID-19 vaccination status, SARS-CoV-2 re-infection status, body mass index (BMI), previous use of other COVID-19 treatments, and a set of comorbidities (diabetes, hypertension, chronic heart diseases, chronic respiratory diseases, moderate/severe renal disease, severe liver disease, solid cancer, haematological disease, immunosuppressive disease or treatment, and solid organ transplant), we did not detect a significant difference in mortality between the two groups (adjusted hazard ratio [aHR] 1.07, 95% CI: 0.96–1.19; Fig. 1). In Scotland, 97 (27.0%) of 359 participants in the sarilumab group died by day 28 compared with 114 (29.4%) of 388 participants in the tocilizumab group (aHR 0.92, 0.68–1.23). By day 90, 930 (23.1%) deaths in the sarilumab group and 1355 (23.7%) deaths in the tocilizumab group occurred in England (aHR 1.01, 0.91–1.13), and 113 (31.5%) deaths in the sarilumab group and 127 (32.7%) deaths in the tocilizumab group occurred in Scotland (aHR 0.92, 0.69–1.21). Cumulative survival curves are provided in the Supplementary Fig. 1, and the Schoenfeld residual plots indicated proportional hazards (Supplementary Fig. 2).

Within 28 days, 2770 (68.8%) participants in the sarilumab group and 3950 (69.2%) participants in the tocilizumab group were discharged from hospital in England (aHR 0.98, 95% CI: 0.91–1.04) and 246 (68.5%) participants in the sarilumab group and 260 (67.0%) participants in the tocilizumab group were discharged in Scotland (aHR 1.07, 95% CI: 0.88–1.29).

**Fig. 2:**
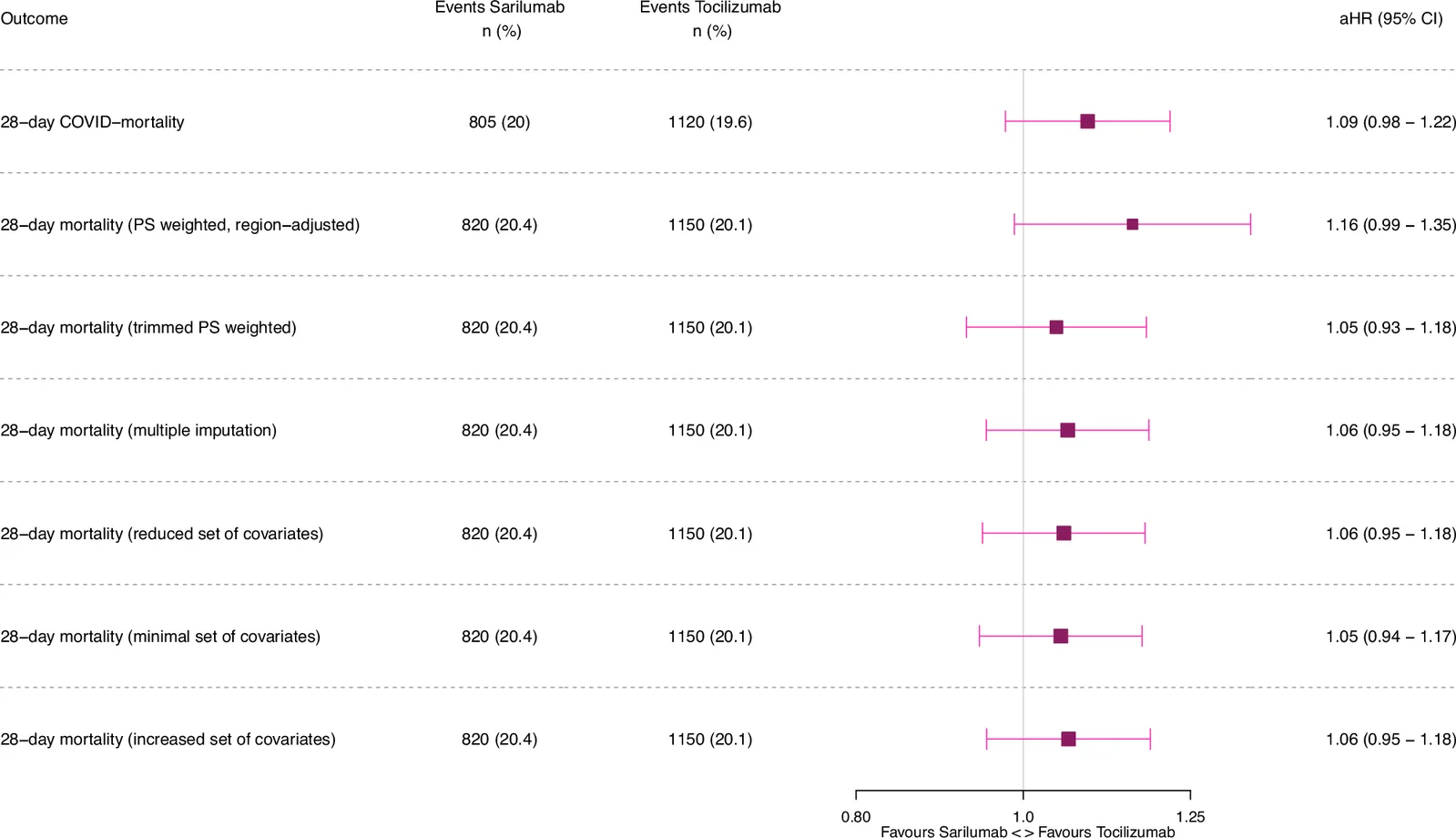
Sensitivity analyses for the primary outcome (OpenSAFELY only). The square represents the point estimate of the adjusted hazard ratio in each sensitivity analysis, with its size proportional to the estimation precision; the error bars represent the corresponding 95% confidence interval. Summary-level source data are provided as a Source data file. aHR adjusted hazard ratio, CI confidence interval, PS propensity score.

In the sensitivity analysis in the OpenSAFELY database, the results of the propensity-score weighted Cox models were consistent with those in the main analyses (Fig. 2). The covariate balance check after weighting the study population based on the propensity score indicated a sufficient balance (standardised mean difference <0.1) between the two groups, except for one region (Supplementary Fig. 3). We therefore (i) additionally adjusted for region in the propensity score outcome model and (ii) trimmed the propensity score at the lower 5th percentile and upper 95th percentile, with the latter model yielding a similar point estimate as in the main results. Results of other sensitivity analyses also suggested comparative effectiveness between the two drugs.

**Fig. 3:**
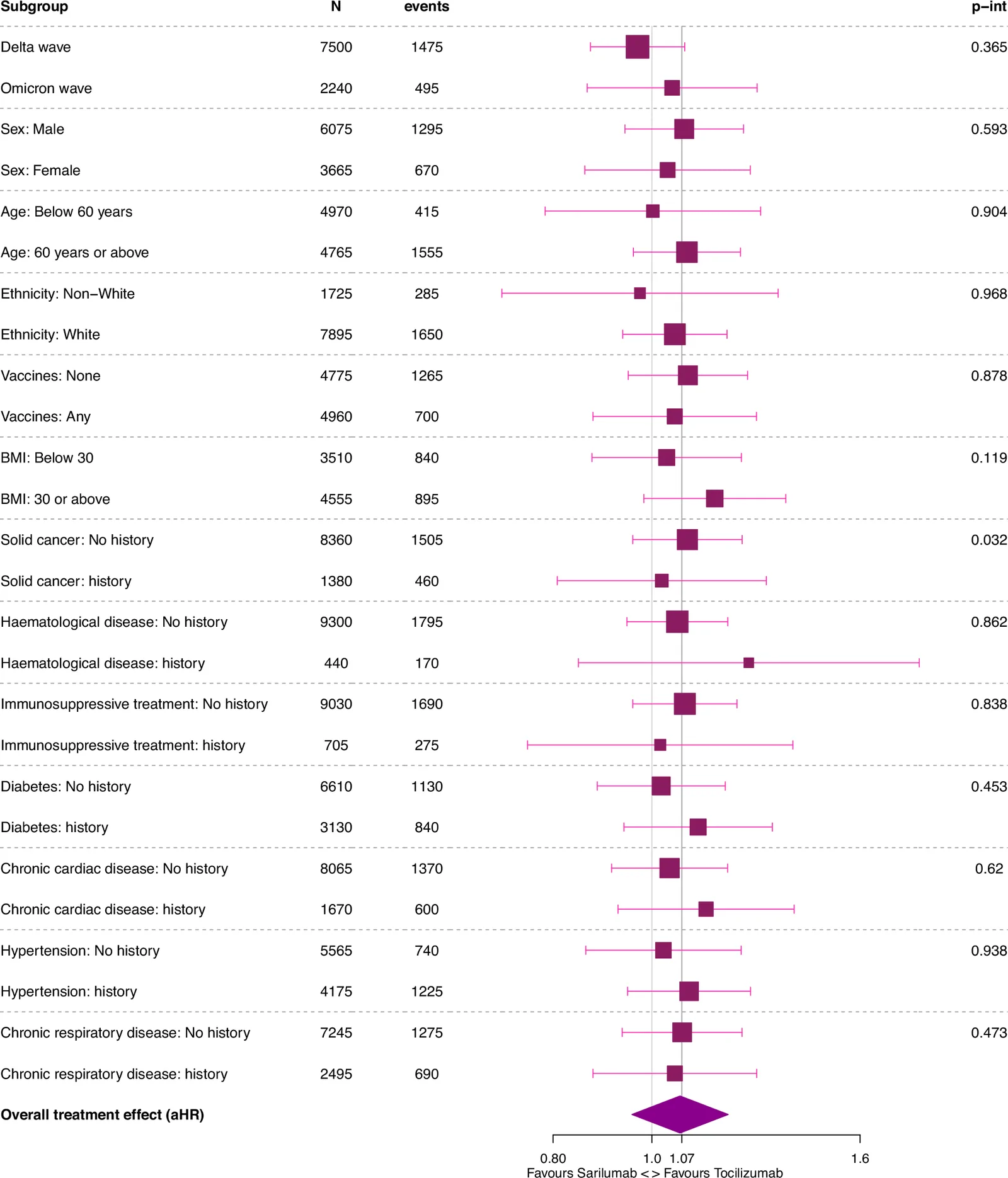
Subgroup analyses for the primary outcome (OpenSAFELY only). The square represents the point estimate of the adjusted hazard ratio in each subgroup, with its size proportional to the statistical precision; the error bars represent the corresponding 95% confidence interval. The centre of the diamond indicates the point estimate of adjusted hazard ratio in the full sample; the width of the diamond represents the 95% confidence interval. Effect modifications were tested using two-sided likelihood ratio tests, with Bonferroni correction applied to address multiple testing. Summary-level source data are provided as a Source data file. *p*-int *p* value for interaction, BMI body mass index, aHR adjusted hazard ratio.

In the subgroup analyses, we did not observe likely effect modification on the comparative effectiveness by any of the pre-specified subgroups (variant of concern, vaccination status, age, sex, ethnicity, BMI, or comorbidities; Fig. 3).

## Discussion

In this large-scale comparative effectiveness study, following the target trial emulation framework and conducted in two separate databases across England and Scotland, we found no significant difference in effectiveness between tocilizumab and sarilumab in terms of mortality or time to hospital discharge among adults hospitalised with severe COVID-19. In addition, we found no credible effect modification by variant of concern, vaccination status, age, sex, ethnicity, BMI, or comorbidities (solid cancer, haematological disease, immunosuppressive treatment, diabetes, hypertension, chronic cardiac disease, and chronic respiratory disease).

This study has a number of important strengths. Firstly, the study period covers a period of clinical equipoise regarding drug effectiveness and thus maximising comparability to a randomised trial. Both OpenSAFELY and EAVE II are sources of high-quality granular data that have enabled research directly influencing clinical decision-making during the pandemic^14,15^. The study is large, and replication of findings from analyses across two UK nations with different data sources adds to the robustness of evidence. We used a standardised protocol following the target trial emulation framework to minimise design-related biases^16^, and findings were robust in multiple sensitivity analyses. Finally, a study within the NHS, where healthcare is free at the point of delivery, limits bias related to inequality in who can access healthcare compared to other global settings.

However, our study has several limitations. First, despite the granular and large-scale data, the possibility of unmeasured residual confounding cannot be ruled out, although there is no evidence to suggest that the type of treatment was influenced by patient characteristics. Second, we were unable to use an identical set of baseline covariates related to treatment indication across the two nations due to lower sample size and the data availability in the EAVE II database, although in the English data, varying the set of confounders—both by including fewer or additional covariates—had only a minor impact on the overall results. Third, the linked hospital episode data records are limited in granularity. As a result, we were unable to fully ascertain all eligibility criteria (e.g. platelet count, hypersensitivity to IL-6 or respiratory support). However, COVID-19 treatment with IL-6 inhibitors was strictly monitored and guided by clinical guidelines in the UK, and therefore, we assumed that receiving an IL-6 inhibitor indicated no contraindication to these. More importantly, for a comparative effectiveness study evaluating two active treatments with identical clinical indications, both of which were available to clinicians during the study period, and used under prevailing clinical equipoise, the absence of such eligibility information is unlikely to introduce systematic bias. Fourth, the days from symptom onset to treatment initiation were not available. However, it is unlikely that the days since symptom onset would have influenced the indication for one IL-6 inhibitor over the other, and both days since positive test and days since hospital admission were short (median of 1 day) and comparable between the two groups.

This study adds to the existing evidence in a number of important ways. Firstly, we were able to conduct a hypothetical target trial that produced similar evidence to the recently published REMAP-CAP trial, the only randomised trial designed to compare tocilizumab with sarilumab^8^. Utilising a Bayesian analytical approach, REMAP-CAP determined that tocilizumab and sarilumab met the criteria for equivalence. Recruitment of the last trial participant happened in April 2021. Our study has extended this evidence base by covering a study period after the emergence of the delta and omicron SARS-CoV-2 variants and including largely vaccinated populations. Previous meta-analyses have synthesised evidence from over 40 randomised trials that evaluated one or both IL-6 inhibitors against standard of care or placebo but were unable to establish the comparative effectiveness of tocilizumab and sarilumab^1,4^. Two subsequent network meta-analyses sought to address this gap by integrating direct and indirect evidence from randomised trials, showing similar effect estimates as our study, but rated the evidence for the comparison as low^6,7^.

The higher mortality observed in the Scottish cohorts when compared to the English cohorts is most likely a reflection of case-mix differences. In particular, higher rates of obesity have consistently been observed across Scotland than in England^17^—a risk factor for severe COVID-19^18^

To date, several clinical guidelines have favoured tocilizumab over sarilumab for the treatment of severe COVID-19—or have excluded sarilumab entirely from treatment recommendations^12^. However, the WHO guidelines^5^ endorse the use of both IL-6 inhibitors interchangeably. Our results, taken together with the results of the REMAP-CAP study, suggest changes to guidelines to align with the WHO recommendation, which provides treatment options even if there are issues with global supply chains of either drug.

In conclusion, among adults hospitalised due to COVID-19, we observed no difference between sarilumab versus tocilizumab with regard to death and hospital discharge, providing real-world evidence for comparable effectiveness of these two treatments.

## Method

### Data sources

This study was conducted in accordance with the ethical principles outlined in the Declaration of Helsinki. The OpenSAFELY project was approved by the Health Research Authority (REC reference 20/LO/0651) and by the LSHTM Ethics Board (reference 21863). Ethical approval for the EAVE II project was obtained from the National Research Ethics Service Committee, Southeast Scotland 02 (reference number: 12/SS/0201), and the Public Benefit and Privacy Panel for Health and Social Care (reference number: 1920-0279).

In England, we used primary care records managed by the General Practitioner (GP) software provider The Phoenix Partnership (TPP), linked to Office of National Statistics (ONS) death registration data, the national coronavirus testing records from the Second Generation Surveillance System, the national vaccine register (National Immunisation Management System), the NHS Secondary Use Service data and the COVID-19 therapeutics dataset through OpenSAFELY. All data is linked, stored and analysed securely using the OpenSAFELY platform, https://www.opensafely.org/, as part of the ongoing NHS England OpenSAFELY COVID-19 service. OpenSAFELY-TPP contains pseudonymised data of approximately 40% of the English population, including coded diagnoses, medications and physiological parameters. No free text data is included. No GP data from patients who have registered a Type-1 Opt out with their GP surgery is included in this study. All code is shared openly for review and re-use under the MIT open license (https://github.com/opensafely/tocilizumab_and_sarilumab). Detailed pseudonymised patient data is potentially re-identifiable and therefore not shared. Previous work has highlighted the high representativeness of OpenSAFELY-TPP to the general population in England (e.g. compared against estimates from the ONS)^19^. Further details of OpenSAFELY information governance are provided in the Supplementary Methods.

In Scotland, we used Early Pandemic Evaluation and Enhanced Surveillance of COVID-19 (EAVE) II, a population health data platform during the COVID-19 pandemic that consolidated linked pseudonymised data across the health system using the Community Health Index number into a near real-time national longitudinal cohort, and included community prescribing (Prescribing Information System), hospital prescribing (Hospital Electronic Prescribing and Medicines Administration [HEPMA]), hospital admissions and episodes (Scottish Morbidity Record [SMR01], Rapid Preliminary Inpatient Data [RAPID], Scottish Intensive Care Society Audit Group database), vaccinations (Turas Vaccine Management Tool), SARS-CoV-2 testing (Electronic Communication of Surveillance Scotland [ECOSS]), and deaths (National Records of Scotland [NRS]). Covering 5.4 million people, approximately 99% of the Scottish population, EAVE II was developed in response to the COVID-19 pandemic^20^.

### Specification of the target trial

We designed this observational comparative effectiveness study to emulate a target trial (i.e. a hypothetical pragmatic trial that would have answered the causal question of interest). The key components of the target trial emulation protocol are summarised in the Supplementary Table 1. We emulated the pragmatic target trial separately across the two nations. The study is reported according to the Transparent Reporting of Observational Studies Emulating a Target Trial (TARGET) guideline^21^.

### Study population

The study population included adults (≥18 years old) who were hospitalised due to COVID-19 and prescribed either tocilizumab or sarilumab between July 1, 2021 and February 28, 2022. In July 2021, these two IL-6 inhibitors started being frequently prescribed for COVID-19 treatment12 and omicron peaked in January/February 2022.18 In OpenSAFELY, COVID-related admission was directly recorded in the COVID-19 therapeutics dataset, while in EAVE II admissions due to COVID-19 were identified as those occurring within 28 days of a positive Reverse Transcription Polymerase Chain Reaction test or those with an International Classification of Diseases, 10th Revision (ICD-10) code for COVID-19 (U07.1 and U07.2) in their admission record (in SMR01 and/or RAPID) as defined in previous studies^22^.

According to NHS clinical guidelines, to be eligible to receive an IL-6 inhibitor for treatment of COVID-19, COVID-19 patients should have been receiving dexamethasone (or an equivalent corticosteroid) unless contraindicated and requiring respiratory support (or having hypoxaemia with evidence of inflammation)^11^. Besides drug-specific hypersensitivity and slightly stricter requirements in terms of liver function and platelet count levels for sarilumab, the clinical eligibility criteria were the same across the two IL-6 treatments. People were excluded if they had (1) <18 or ≥110 years of age, (2) missing information on sex or region, or (3) recorded as having received tocilizumab and sarilumab on the same date.

### Interventions of interest

The interventions of interest were the first record of treatment with tocilizumab or sarilumab between July 1, 2021 and February 28, 2022. In OpenSAFELY, this was ascertained from the COVID-19 therapeutics dataset based on the national Blueteq system, while in EAVE II, this was ascertained from HEPMA as defined in prior research^23^. Both treatment datasets summarised therapeutics specifically to treat COVID-19 and no other condition.

### Outcomes and follow-up

The primary outcome was all-cause mortality within 28 days after treatment initiation, extracted from the ONS mortality database in OpenSAFELY and from the NRS mortality database in EAVE II. Secondary outcomes included 90-day all-cause mortality and time to hospital discharge since treatment initiation. The date of tocilizumab or sarilumab prescription was defined as the baseline date. People were then followed from their baseline date until the earliest of either reaching the outcome, death (when analysing time to hospital discharge), or the end of the follow-up period.

### Covariates

To mimic the randomisation of the target trial, we assumed randomisation conditional on the following baseline covariates: age (restricted cubic splines), sex (biological attribute, as reported in the electronic health records), NHS region, calendar time (restricted cubic splines), ethnicity (grouped into five broad categories: White, Black or Black British, Asian or Asian British, Mixed, Other), (Scottish) Index of Multiple Deprivation ([(S)IMD], as quintiles derived from the patient’s postcode at lower super output area level), COVID-19 vaccination status (un-vaccinated, one vaccination, two vaccinations, or three or more), SARS-CoV-2 re-infection status (positive test or clinical diagnosis code or exposure to COVID-19 drug at least 3 months prior), BMI, most recent record, grouped into <25.0, 25.0 to <30.0, 30.0 to <35, and ≥35.0 kg/m^2^, previous use of other COVID-19 treatments (remdesivir, casirivimab/imdevimab and sotrovimab), diabetes, hypertension, chronic heart diseases, chronic respiratory diseases, moderate/severe renal disease, severe liver disease, solid cancer, haematological disease, immunosuppressive disease or treatment, and solid organ transplant. These covariates of interest, potentially prognostic for treatment initiation and outcome, were identified through literature review and discussions with domain experts. Comorbidities were identified through SNOMED CT codes in primary care records and ICD-10 in secondary care records. Ethnicity was identified through SNOMED CT codes and supplemented with information from secondary care records. Individuals with missing BMI, ethnicity, and (S)IMD were included with a missing indicator to maximise power, but alternative assumptions were tested in sensitivity analyses. Absence of recorded codes in terms of comorbidities, vaccination, re-infection, and prior COVID-19 treatment was assumed as not having such an event. Considering the sample size and data availability in the EAVE II database, the following baseline covariates were excluded for the EAVE II trial emulation: SARS-CoV-2 re-infection status, previous use of other COVID-19 treatments, solid cancer, immunosuppressive disease or treatment, and solid organ transplant.

### Statistical analysis

Cox proportional hazards models, with follow-up time as the time scale, were used to estimate hazard ratios (HR) and 95% confidence intervals (CI) for the association between treatment and each outcome in turn, adjusted for the above-mentioned covariates. In the OpenSAFELY analysis, the Cox model was stratified by NHS region to account for the geographical heterogeneity in COVID-19 outcomes. For the secondary outcome ‘time to discharge within 28 days’, we assumed death as a competing risk, censored them and assumed the worst case (i.e. no discharge until the end of the 28-day follow-up)—as recommended and applied in corresponding trial analyses^24,25^. We used Schoenfeld residual plots to assess the proportional hazards assumption.

We conducted several sensitivity analyses: (1) We explored a reduced set of baseline covariates for the conditional randomisation (age, sex, calendar time, ethnicity, IMD, COVID-19 vaccination status and SARS-CoV-2 re-infection status) and a minimal set (age, sex and calendar time). (2) We extended the conditional randomisation to additionally include rural/urban area, days between last COVID-19 vaccination and treatment initiation, and days between hospital admission and treatment initiation. (3) We used a propensity score weighted Cox model with robust variance estimators to mimic the randomisation instead of using covariate adjustment. The propensity score was derived from a logistic regression modelling the conditional probability of being treated with tocilizumab based on all baseline covariates. We conducted a covariate balance check after weighting using standardised mean differences between the two groups and a threshold of <0.10 as the indicator for being balanced. (4) To explore the impact of missing data in IMD, BMI and ethnicity, we conducted multiple imputation using chained equations techniques^26^. (5) We assessed the association on COVID-19-related deaths only. These deaths were defined as a death whereby the underlying or contributory cause on the death certificate (ONS mortality database) was COVID-19 (ICD-10 codes U07.1, U07.2). As per protocol, we had planned to conduct a Bayesian Cox regression as additional sensitivity analysis to inform the evidence in favour of or against the null hypothesis. However, due to the failed convergence of the model, we had to drop this analysis.

We conducted several subgroup analyses to assess potential effect modifications: Dominant circulating variant of concern (delta variant before December 6, 2021, versus omicron BA.1 variant thereafter), COVID-19 vaccination status (none versus one or more vaccinations), age group (below 60 vs 60 years and above), sex (female versus male), ethnicity (white versus non-white), BMI (below 30 versus 30 or above), and presence versus absence of comorbidities (solid cancer, haematological disease, immunosuppressive treatment, diabetes, hypertension, chronic cardiac disease, and chronic respiratory disease). Effect modifications were tested using two-sided likelihood ratio tests, with Bonferroni correction applied to address multiple testing.

When presenting the results from the OpenSAFELY database, all counts of 7 or below were redacted, and the counts above 7 were rounded to the nearest five to minimise potential disclosure. Data management was performed using Python 3.10, with analysis carried out using Stata 16.1 and R v4.5.0.

### Reporting summary

Further information on research design is available in the Nature Portfolio Reporting Summary linked to this article.

## Supplementary information

Supplementary Information

Reporting Summary

Transparent Peer Review file

## Source data

Source data

## Data Availability

All data were linked, stored and analysed securely within the OpenSAFELY platform (https://opensafely.org/) and the EAVE II platform (https://usher.ed.ac.uk/eave-ii). Data include pseudonymised data such as coded diagnoses, medications and physiological parameters. No free text data are included. Detailed pseudonymised patient data are potentially re-identifiable and therefore not shared. Data can be analysed through the OpenSAFELY and EAVE II platforms, subject to appropriate agreement, approvals and training as detailed in the platform websites. Summary-level Source data are provided with this paper.

## Code availability

Code for data management and analysis in OpenSAFELY, the codelists and the pre-specified protocol are archived online (https://github.com/opensafely/tocilizumab_and_sarilumab/tree/main), and all public logs of analysis activity are available online (https://jobs.opensafely.org/effectiveness-and-safety-of-covid-19-treatments-for-hospitalised-patients/tocilizumab_sarilumab/releases/).
