# Author Correction: Tocilizumab versus sarilumab among adults hospitalised with COVID-19: target trial emulation across England and Scotland

**DOI:** 10.1038/s41467-026-78874-2

**Published:** 2026-11-04

**Authors:** Bang Zheng, Amanj Kurdi, Alain Amstutz, Amelia Green, Emily Herrett, John Tazare, Qing Wen, Viyaasan Mahalingasivam, Rebecca Smith, Brian MacKenna, Amir Mehrkar, Seb Bacon, Ben Goldacre, Chris Robertson, Aziz Sheikh, Laurie Tomlinson

**Affiliations:** 1 Department of Epidemiology & Biostatistics, School of Public Health, Peking University, Beijing, China; 2 Key Laboratory of Epidemiology of Major Diseases (Peking University), Ministry of Education, Beijing, China; 3 Department of Non-communicable Disease Epidemiology, London School of Hygiene and Tropical Medicine, London, UK; 4 Peking University Center for Public Health and Epidemic Preparedness & Response, Beijing, China; 5 Strathclyde Institute of Pharmacy and Biomedical Sciences, University of Strathclyde, Glasgow, UK; 6 Public Health Scotland, Edinburgh, UK; 7 College of Pharmacy, Hawler Medical University, Erbil, Iraq; 8 Al-Kitab University, Kirkuk, Iraq; 9 Department of Public Health Pharmacy and Management, School of Pharmacy, Sefako Makgatho Health Sciences University, Pretoria, South Africa; 10 Electronic Health Records Group, Population Health Sciences, Bristol Medical School, University of Bristol, Bristol, UK; 11 Oslo Center for Biostatistics and Epidemiology, Oslo University Hospital, Oslo, Norway; 12 Division Clinical Epidemiology, Department of Clinical Research, University Hospital Basel and University of Basel, Basel, Switzerland; 13 Nuffield Department of Primary Care Health Sciences, University of Oxford, Oxford, UK; 14 Department of Medical Statistics, London School of Hygiene and Tropical Medicine, London, UK; 15 Centre for Public Health, School of Medicine, Dentistry and Biomedical Sciences, Queen’s University Belfast, Belfast, UK; 16 Department of Mathematics and Statistics, University of Strathclyde, Glasgow, UK; 17 Usher Institute, University of Edinburgh, Edinburgh, UK

**Keywords:** Viral infection, Therapeutics, Respiratory signs and symptoms

Correction to: *Nature Communications* https://doi.org/10.1038/s41467-026-73134-9, published online 15 May 2026

In this article the author’s name Aziz Sheikh was written as Sir Aziz Sheikh. The original article has been updated.

