# Supplementary material for: Tocilizumab versus sarilumab among adults hospitalised with COVID-19: target trial emulation across England and Scotland
Source: Nat Commun. 2026 May 15;17:6453. doi: 10.1038/s41467-026-73134-9 (PMC13376390; doi:10.1038/s41467-026-73134-9)
Supplement: Supplementary file 1 — Supplementary Information [file 41467_2026_73134_MOESM1_ESM.pdf]

Supplementary Information

Tocilizumab versus sarilumab among adults hospitalised with COVID-19: target trial emulation across England and Scotland

Supplementary Methods

Information governance and ethical approval

OpenSAFELY: NHS England is the data controller of the NHS England OpenSAFELY COVID-19 Service; TPP is the data processor; all study authors using OpenSAFELY have the approval of NHS England. This implementation of OpenSAFELY is hosted within the TPP environment which is accredited to the ISO 27001 information security standard and is NHS IG Toolkit compliant. Patient data has been pseudonymised for analysis and linkage using industry standard cryptographic hashing techniques; all pseudonymised datasets transmitted for linkage onto OpenSAFELY are encrypted; access to the NHS England OpenSAFELY COVID-19 service is via a virtual private network (VPN) connection; the researchers hold contracts with NHS England and only access the platform to initiate database queries and statistical models; all database activity is logged; only aggregate statistical outputs leave the platform environment following best practice for anonymisation of results such as statistical disclosure control for low cell counts.

The service adheres to the obligations of the UK General Data Protection Regulation (UK GDPR) and the Data Protection Act 2018. The service previously operated under notices initially issued in February 2020 by the the Secretary of State under Regulation 3(4) of the Health Service (Control of Patient Information) Regulations 2002 (COPI Regulations), which required organisations to process confidential patient information for COVID-19 purposes; this set aside the requirement for patient consent. As of 1 July 2023, the Secretary of State has requested that NHS England continue to operate the Service under the COVID-19 Directions 2020. In some cases of data sharing, the common law duty of confidence is met using, for example, patient consent or support from the Health Research Authority Confidentiality Advisory Group.

Taken together, these provide the legal bases to link patient datasets using the service. GP practices, which provide access to the primary care data, are required to share relevant health information to support the public health response to the pandemic, and have been informed of how the service operates. This study was approved by the Health Research Authority (REC reference 20/LO/0651) and by the LSHTM Ethics Board (reference 21863).

EAVE II: Data are held by Public Health Scotland and were made available under the Public Health Scotland COVID-19 data governance framework. As such, individual-level patient data are not publicly available. Access to data was granted following appropriate ethical and governance approvals. Ethical approval for EAVE II was obtained from the National Research Ethics Service Committee, Southeast Scotland 02 (reference number: 12/SS/0201), and the Public Benefit and Privacy Panel for Health and Social Care (reference number: 1920-0279).

Supplementary Table 1: Target trial specification and emulation, following the TARGET reporting guideline and template

**Protocol element**	**Hypothetical pragmatic randomized trial (target trial)**	**Target trial emulation**, done separately for OpenSAFELY and EAVE II	**Notes**
**Eligibility criteria**	<u>Inclusion criteria:</u><br>1) Aged 18-110 years at enrolment<br>2) Hospitalised due to COVID-19<br>3) Receiving dexamethasone or an equivalent corticosteroid unless contraindicated<br>4) Requiring respiratory support or having hypoxaemia with evidence of inflammation<br><br><u>Exclusion criteria:</u><br>1) Hypersensitivity to IL-6 inhibitors<br>2) Baseline ALT/AST >5x ULN<br>3) Platelet count <150 x 10^3/µl	*Same as for the target trial, except:*<br>- In OpenSAFELY: COVID-related admissions were directly extracted from the COVID-19 therapeutics dataset<br>- In EAVE II: COVID-related admissions were identified as those occurring within 28 days of a positive RT-PCR test or those hospital admission with an ICD-10 code for COVID-19 (U07.1 and U07.2) in their admission record (in SMR01 and/or RAPID)<br>- Data on respiratory support, corticosteroid use and listed exclusion criteria are not consistently available across the two data platforms, but we assumed that receiving an IL-6 indicated there was no contraindication to these	In addition, we applied data quality assurance criteria: We excluded individuals with a) missing information on sex or region, and b) who received tocilizumab and sarilumab on the same date
**Treatment strategies**	Eligible participants are randomised to either:<br>1. Tocilizumab: a single 60-minute intravenous infusion of 8 mg/kg<br>2. Sarilumab: a single 60-minute intravenous infusion of 400 mg	*Same as for the target trial*	We only considered the first prescription to any of the two treatments.<br>In OpenSAFELY, this was ascertained from the COVID-19 therapeutics dataset based on the national Blueteq system, while in EAVE II this was ascertained from HEPMA.
**Assignment procedures**	Eligible participants are randomly assigned to one of the two groups, and are aware of which group they were assigned to (open-label).	We assumed randomisation conditional on the following baseline covariates: age, sex, region, calendar time, ethnicity (grouped into five broad categories: White, Black or Black British, Asian or Asian British, Mixed, Other), Index of Multiple Deprivation ([IMD], as quintiles derived from the patient's postcode at lower super output area level), COVID-19 vaccination status (unvaccinated, one vaccination, two vaccinations, or three or more), SARS-CoV-2 re-infection status (positive test or clinical diagnosis code or exposure to COVID-19 drug at least three months prior), body mass index ([BMI], most recent record), previous use of other COVID-19 treatments, diabetes, hypertension, chronic heart diseases, chronic respiratory diseases, moderate/severe renal disease, severe liver disease, solid cancer, hematological disease, immunosuppressive disease or treatment, and solid organ transplant.	Covariates were identified based on previous literature, discussions with domain experts and data availability in each database.<br><br>Comorbidities were extracted through SNOMED CT codes in primary care records and ICD-10 in secondary care records. Ethnicity was extracted through SNOMED CT codes and supplemented with information from secondary care records.<br><br>Considering the sample size and data availability in the EAVE II database, the following baseline covariates were excluded in EAVE II: SARS-CoV-2 re-infection status, previous use of other COVID-19 treatments, solid cancer, immunosuppressive disease or treatment, and solid organ transplant

**Follow-up**	Primary:<br>- Each participant will be followed up until death, or end of 28-day follow-up, whichever occurs first.<br>Secondary:<br>- 90-day mortality: Same as for primary endpoint<br>- Time to hospital discharge: Each participant will be followed up until hospital discharge, death, or end of 28-day follow-up, whichever occurs first.	*Same as for the target trial*	
**Outcomes**	Primary:<br>- All-cause mortality within 28 days<br>Secondary:<br>- All-cause mortality within 90 days<br>- Time to hospital discharge within 28 days	*Same as for the target trial*	Mortality data extracted from the ONS mortality database in OpenSAFELY and from the NRS mortality database in EAVE II.<br>Time to hospital discharge extracted from the linked secondary care records.
**Causal contrast**	Intention-to-treat effect<br><br>Per-protocol effect, i.e., effect of receiving 1 dose of tocilizumab vs receiving 1 dose of sarilumab among adults hospitalised due to COVID-19 between July 1, 2021 and February 28, 2022.<br><br>We will measure the effect in hazard ratios (HR) and 95% confidence intervals (CI).<br><br>For the time to discharge outcome, deaths will be treated as a censoring event with worst outcome imputation, to account for the competing risk, which targets a sub-distribution hazard estimand: "Among the full cohort, what is the effect of treatment on the probability of being discharged over time, accounting for the fact that some die?"	No intention-to-treat effect estimated<br><br>Observational analogue of the per-protocol effect: "Effect of receiving 1 dose of tocilizumab vs receiving 1 dose of sarilumab among adults hospitalised due to COVID-19 between July 1, 2021 and February 28, 2022"<br><br>We measured the effect in hazard ratios (HR) and 95% confidence intervals (CI).<br><br>For the time to discharge outcome, deaths were treated as a censoring event with worst outcome imputation, to account for the competing risk, which targets a sub-distribution hazard estimand: "Among the full cohort, what is the effect of treatment on the probability of being discharged over time, accounting for the fact that some die?"	Only information on treatment prescription is available, therefore, we estimate a per-protocol effect. However, in the case of an intravenous in-hospital treatment among severely ill patients it is reasonable to assume that everyone received the intended treatment and thus this closely corresponds to an intention-to-treat effect.<br><br>Handling death as worst-case imputation for time to discharge analyses has been recommended previously, e.g.<br>Li H, Gleason KJ, Hu Y, et al. Handling death as an intercurrent event in time to recovery analysis in COVID-19 treatment clinical trials. *Contemp Clin Trials.* 2022 Aug;119:106758.
**Identifying assumptions**	(1) "Interventions are well-defined": Given by the fact of a prospective randomized trial following a pre-specified protocol clarifying the interventions<br>(2) "Exchangeability (no unmeasured confounding at baseline)": Given by randomization<br>(3) "Positivity assumption": Given by design, i.e., every eligible person is randomized and the randomization algorithm assigns each participant to each arm with positive probability<br>(4) "Conditional exchangeability in per protocol analysis (no selection bias	(1) "Interventions are well-defined": Given by the fact that this is a comparative effectiveness study comparing two active well-defined treatments with clear clinical treatment protocols for the treatment of COVID-19.<br>(2) "Exchangeability (no unmeasured confounding at baseline)": We assumed exchangeability by an extensive set of pre-specified clinical, sociodemographic and socioeconomic characteristics (see details in row "Assignment procedures")<br>(3) "Positivity assumption": We assumed that only individuals eligible to receive an IL-6 inhibitor received an IL-6 inhibitor since there were strict clinical inclusion/exclusion criteria in the treatment	

	introduced during follow-up, due to adherence, lost-to-follow-up, or competing events)": For a study among severely-ill patients, at the hospital, for a one-time intravenous treatment, with a short follow-up and mortality and discharge as outcomes, we don't expect any lost-to-follow-up and initiation of the assigned treatment and adherence to it are practically guaranteed. However, for the discharge outcome, death will be considered as a competing event and the focus will be on the cumulative incidence function for discharge.	guidelines (esp. for these expensive treatments). There was clinical equipoise between the two treatments and both available at the time.<br>(4) "Conditional exchangeability in per protocol analysis (no selection bias introduced during follow-up, due to adherence, lost-to-follow-up, or competing events)": For a study among severely-ill patients, at the hospital, for a one-time intravenous treatment, with a short follow-up and mortality and discharge as outcomes, we did not expect any lost-to-follow-up and initiation of the assigned treatment and adherence to it are practically guaranteed. However, for the discharge outcome, death is considered as a competing event and the focus is on the cumulative incidence function for discharge.	
**Data analysis**	Hazard ratios comparing the two groups, using a Cox model with follow-up time as the time scale and stratified by region.<br><br>Subgroup analyses to assess treatment interactions: Dominant circulating variant of concern (delta variant before December 6, 2021, versus omicron BA.1 variant thereafter), COVID-19 vaccination status (none versus one or more vaccinations), age group (below 60 vs 60 years and above), sex (female versus male), ethnicity (white versus non-white), BMI (below 30 versus 30 or above), and presence versus absence of comorbidities (solid cancer, hematological disease, immunosuppressive treatment, diabetes, hypertension, chronic cardiac disease, and chronic respiratory disease). Effect modifications by these subgroups will be tested using likelihood ratio tests, with Bonferroni correction applied to address multiple testing.	*Same as for the target trial, except:*<br>adjusting for the above-mentioned covariates in the Cox model (see row "Assignment procedures").<br><br>Subgroup analyses/effect modification was done the same way as described for the target trial.<br><br>We conducted several sensitivity analyses to test several of our assumptions: (1) We explored a reduced set of baseline covariates for the conditional randomisation (age, sex, calendar time, ethnicity, IMD, COVID-19 vaccination status and SARS-CoV-2 re-infection status) and a minimal set (age, sex and calendar time). (2) We extended the conditional randomisation to additionally include rural/urban area, days between last COVID-19 vaccination and treatment initiation, and days between hospital admission and treatment initiation. (3) We used a propensity score weighted Cox model with robust variance estimators to mimic the randomisation instead of using covariate adjustment. The propensity score was derived from a logistic regression modelling the conditional probability of being treated with tocilizumab based on all baseline covariates. We conducted a covariate balance check after weighting using standardised mean differences between the two groups and a threshold of <0.10 as the indicator for being balanced. (4) To explore the impact of missing data in IMD, BMI and ethnicity, we conducted multiple imputation using chained equations techniques. (5) We assessed the association on COVID-19 related deaths only. These deaths were defined as a death whereby the underlying or contributory cause on the death certificate (ONS mortality database) was COVID-19 (ICD-10 codes U07.1, U07.2).	

Supplementary Figure 1: Survival curves for mortality

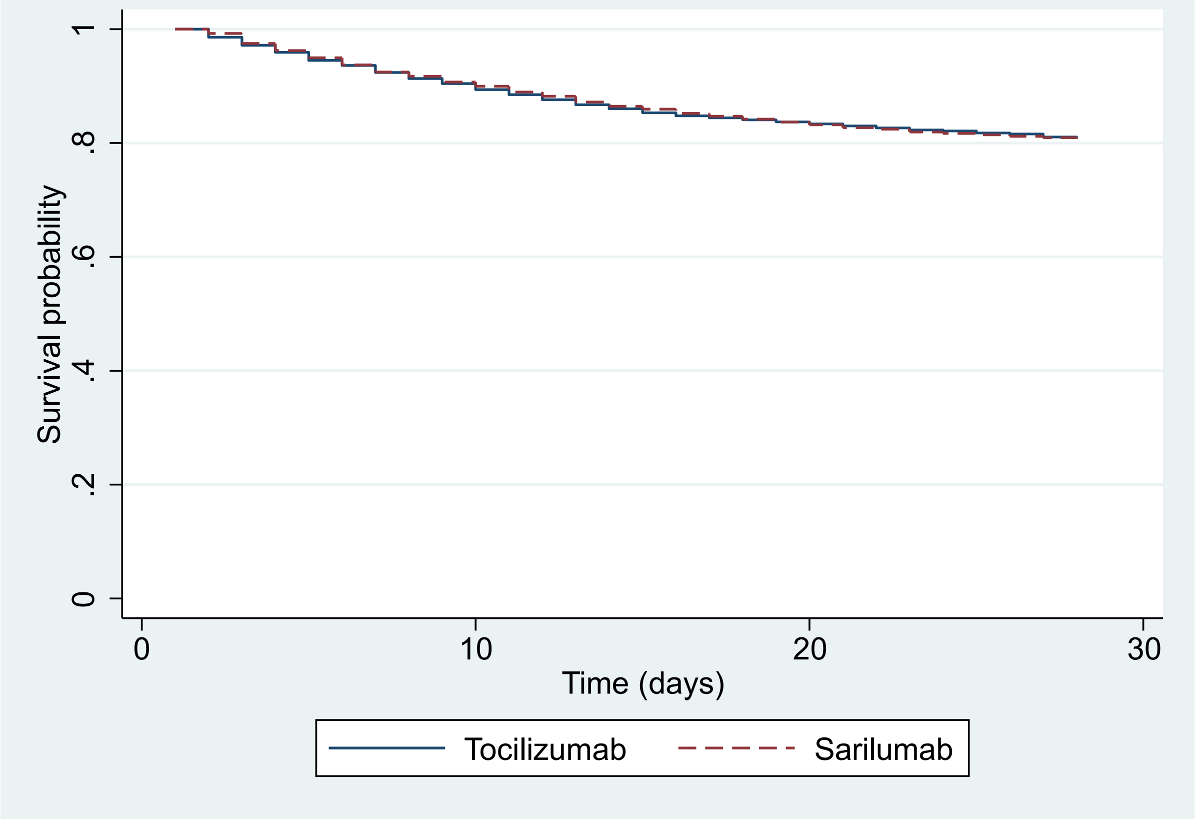

Note: This figure presents the Kaplan-Meier survival curves by drug group in the OpenSAFELY database (N=9740). In accordance with the platform's statistical disclosure control policies, the steps in the Kaplan-Meier curves were delayed until a minimum event count of 10 deaths was accumulated, thereby ensuring a low risk of disclosure.

Supplementary Figure 2: Schoenfeld residual plot for mortality

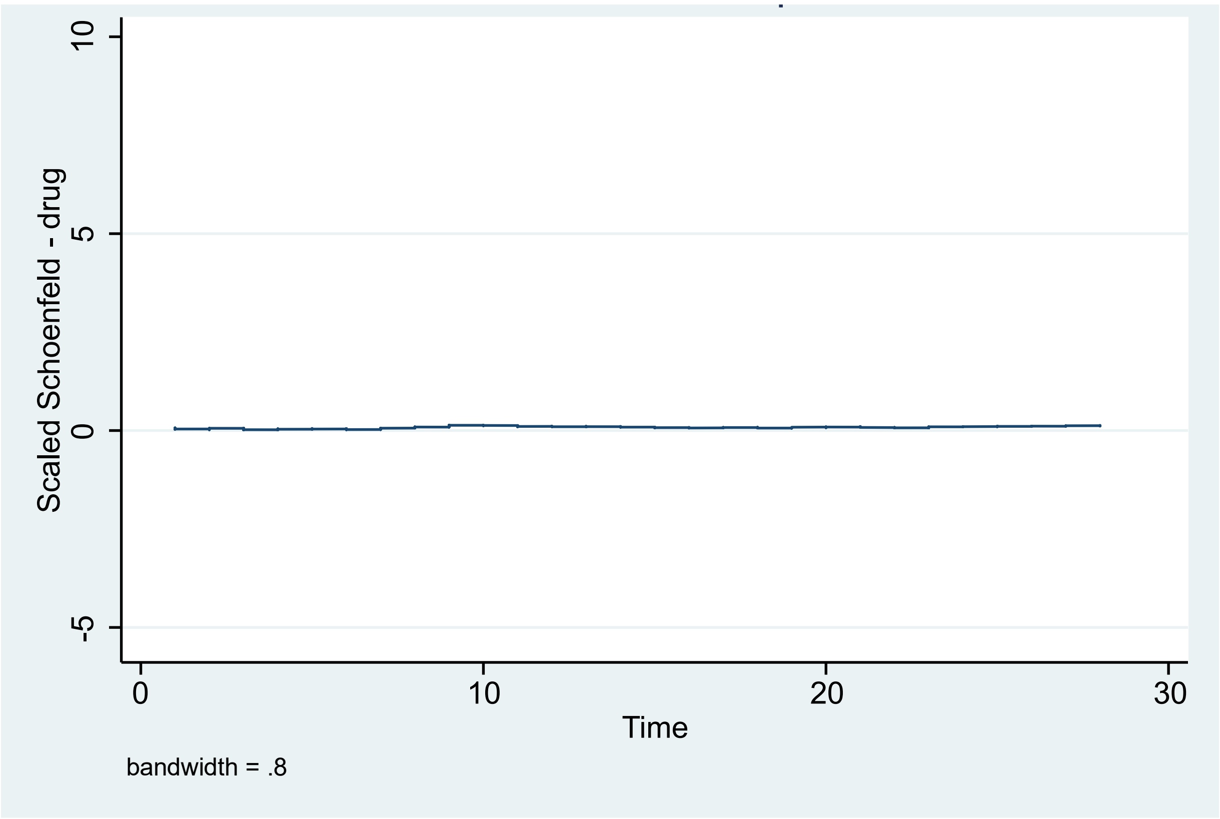

Note: This figure presents the loess smoothed curve of weighted Schoenfeld residuals of drug group across follow-up time in the OpenSAFELY database (N=9740). In accordance with the platform's statistical disclosure control policies, the individual scatter points have been removed.

Supplementary Figure 3: Love Plot: Standardized mean differences before and after weighting

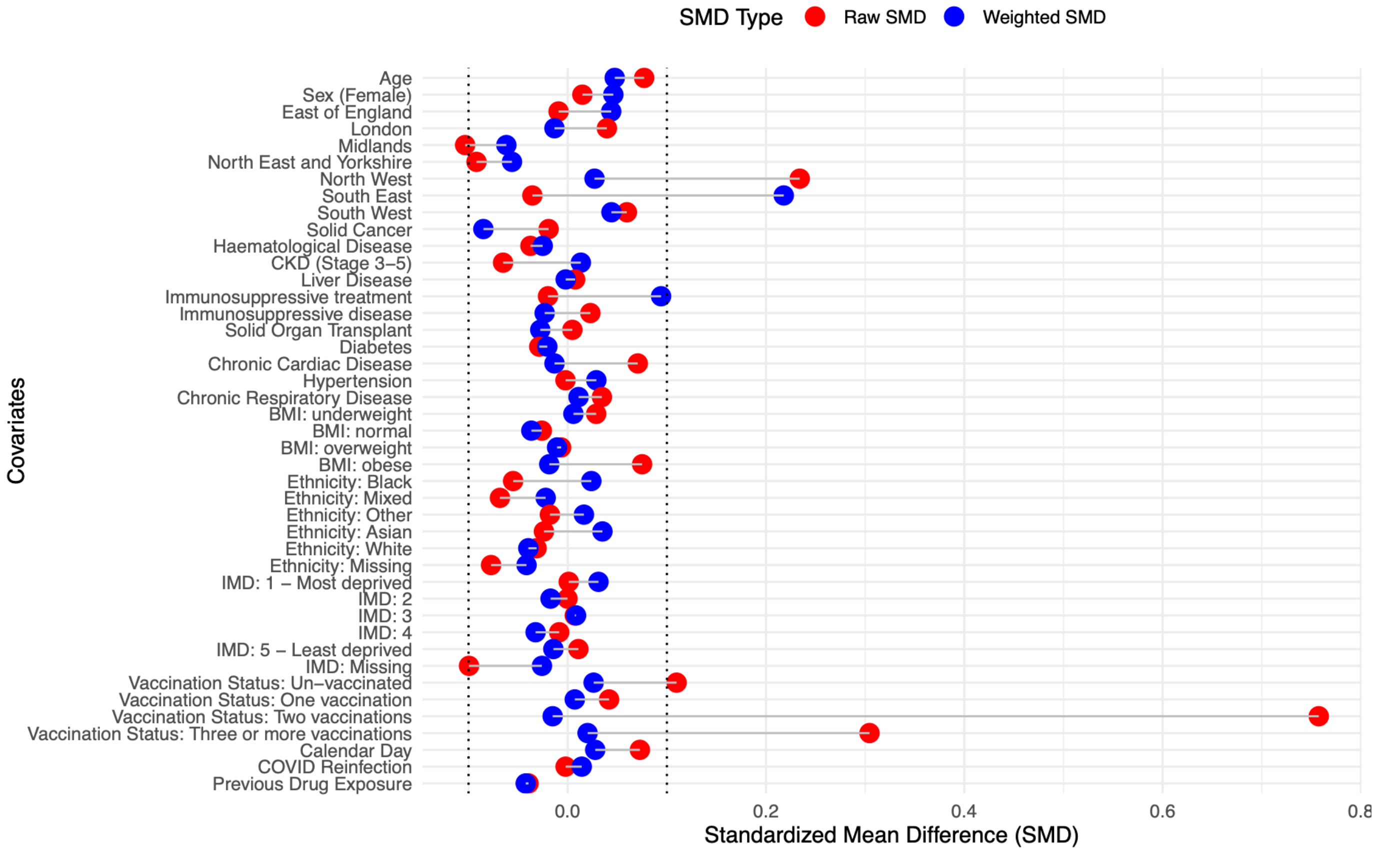

Note: This figure presents the standardized mean differences of covariates between the two drug groups before and after propensity score weighting in the OpenSAFELY database (N=9740).
