# Supplementary material for: Tocilizumab versus sarilumab among adults hospitalised with COVID-19: target trial emulation across England and Scotland
Source: Nat Commun. 2026 May 15;17:6453. doi: 10.1038/s41467-026-73134-9 (PMC13376390; doi:10.1038/s41467-026-73134-9)
Supplement: Supplementary file 2 — Reporting Summary [file 41467_2026_73134_MOESM2_ESM.pdf]

nature portfolio

Corresponding author(s): Sir Aziz Sheikh, Laurie Tomlinson

Last updated by author(s): Mar 28, 2026

Reporting Summary

Nature Portfolio wishes to improve the reproducibility of the work that we publish. This form provides structure for consistency and transparency in reporting. For further information on Nature Portfolio policies, see our Editorial Policies and the Editorial Policy Checklist.

Statistics

For all statistical analyses, confirm that the following items are present in the figure legend, table legend, main text, or Methods section.

n/a	Confirmed	
☐	☒	The exact sample size (*n*) for each experimental group/condition, given as a discrete number and unit of measurement
☒	☐	A statement on whether measurements were taken from distinct samples or whether the same sample was measured repeatedly
☐	☒	The statistical test(s) used AND whether they are one- or two-sided<br>*Only common tests should be described solely by name; describe more complex techniques in the Methods section.*
☐	☒	A description of all covariates tested
☐	☒	A description of any assumptions or corrections, such as tests of normality and adjustment for multiple comparisons
☐	☒	A full description of the statistical parameters including central tendency (e.g. means) or other basic estimates (e.g. regression coefficient) AND variation (e.g. standard deviation) or associated estimates of uncertainty (e.g. confidence intervals)
☐	☒	For null hypothesis testing, the test statistic (e.g. *F*, *t*, *r*) with confidence intervals, effect sizes, degrees of freedom and *P* value noted<br>*Give P values as exact values whenever suitable.*
☒	☐	For Bayesian analysis, information on the choice of priors and Markov chain Monte Carlo settings
☒	☐	For hierarchical and complex designs, identification of the appropriate level for tests and full reporting of outcomes
☒	☐	Estimates of effect sizes (e.g. Cohen's *d*, Pearson's *r*), indicating how they were calculated

Our web collection on statistics for biologists contains articles on many of the points above.

Software and code

Policy information about availability of computer code

Data collection

In England, we used primary care records managed by the General Practitioner (GP) software provider The Phoenix Partnership (TPP), linked to Office of National Statistics (ONS) death registration data, the national coronavirus testing records from the Second Generation Surveillance System (SGSS), the national vaccine register (National Immunisation Management System [NIMS]), the NHS Secondary Use Service (SUS) data and the COVID-19 therapeutics dataset through OpenSAFELY. All data is linked, stored and analysed securely using the OpenSAFELY platform, https://www.opensafely.org/, as part of the ongoing NHS England OpenSAFELY COVID-19 service. OpenSAFELY-TPP contains pseudonymised data of approximately 40% of the English population, including coded diagnoses, medications and physiological parameters. No free text data is included. No GP data from patients who have registered a Type-1 Opt out with their GP surgery is included in this study. All code is shared openly for review and re-use under MIT open license (https://github.com/opensafely/tocilizumab_and_sarilumab).
In Scotland, we used Early Pandemic Evaluation and Enhanced Surveillance of COVID-19 (EAVE) II (https://usher.ed.ac.uk/eave-ii), a population health data platform during the COVID-19 pandemic that consolidated linked pseudonymised data across the health system using the Community Health Index number into a near real-time national longitudinal cohort, and included community prescribing (Prescribing Information System [PIS]), hospital prescribing (Hospital Electronic Prescribing and Medicines Administration [HEPMA]), hospital admissions and episodes (Scottish Morbidity Record [SMR01], Rapid Preliminary Inpatient Data [RAPID], Scottish Intensive Care Society Audit Group database [SICSAG]), vaccinations (Turas Vaccine Management Tool [TVMT]), SARS-CoV-2 testing (Electronic Communication of Surveillance Scotland [ECOSS]), and deaths (National Records of Scotland [NRS]).

Data analysis

Data management was performed using Python 3.10, with analysis carried out using Stata 16.1 and R v4.5.0.

Cox proportional hazards models, with follow-up time as the time scale, were used to estimate hazard ratios (HR) and 95% confidence intervals (CI) for the association between treatment and each outcome in turn, adjusted for the above-mentioned covariates. In the OpenSAFELY analysis, the Cox model was stratified by NHS region to account for the geographical heterogeneity in COVID-19 outcomes. For

the secondary outcome 'time to discharge within 28 days', we assumed death as a competing risk, censored them and assumed the worst case (i.e., no discharge until end of 28-day follow-up) - as recommended and applied in corresponding trial analyses.
We conducted several sensitivity analyses: (1) We explored a reduced set of baseline covariates for the conditional randomisation (age, sex, calendar time, ethnicity, IMD, COVID-19 vaccination status and SARS-CoV-2 re-infection status) and a minimal set (age, sex and calendar time). (2) We extended the conditional randomisation to additionally include rural/urban area, days between last COVID-19 vaccination and treatment initiation, and days between hospital admission and treatment initiation. (3) We used a propensity score weighted Cox model with robust variance estimators to mimic the randomisation instead of using covariate adjustment. The propensity score was derived from a logistic regression modelling the conditional probability of being treated with tocilizumab based on all baseline covariates. We conducted a covariate balance check after weighting using standardised mean differences between the two groups and a threshold of <0.10 as the indicator for being balanced. (4) To explore the impact of missing data in IMD, BMI and ethnicity, we conducted multiple imputation using chained equations techniques. (5) We assessed the association on COVID-19 related deaths only. These deaths were defined as a death whereby the underlying or contributory cause on the death certificate (ONS mortality database) was COVID-19 (ICD-10 codes U07.1, U07.2).

We conducted several subgroup analyses to assess potential effect modifications: Dominant circulating variant of concern (delta variant before December 6, 2021, versus omicron BA.1 variant thereafter), COVID-19 vaccination status (none versus one or more vaccinations), age group (below 60 vs 60 years and above), sex (female versus male), ethnicity (white versus non-white), BMI (below 30 versus 30 or above), and presence versus absence of comorbidities (solid cancer, hematological disease, immunosuppressive treatment, diabetes, hypertension, chronic cardiac disease, and chronic respiratory disease). Effect modifications were tested using likelihood ratio tests, with Bonferroni correction applied to address multiple testing.

For manuscripts utilizing custom algorithms or software that are central to the research but not yet described in published literature, software must be made available to editors and reviewers. We strongly encourage code deposition in a community repository (e.g. GitHub). See the Nature Portfolio guidelines for submitting code & software for further information.

Data

Policy information about availability of data

All manuscripts must include a data availability statement. This statement should provide the following information, where applicable:

- Accession codes, unique identifiers, or web links for publicly available datasets
- A description of any restrictions on data availability
- For clinical datasets or third party data, please ensure that the statement adheres to our policy

All data were linked, stored and analysed securely within the OpenSAFELY platform (https://opensafely.org/) and the EAVE II platform (https://usher.ed.ac.uk/eave-ii). Data include pseudonymised data such as coded diagnoses, medications and physiological parameters. No free text data are included. Detailed pseudonymised patient data is potentially re-identifiable and therefore not shared. Data can be analysed through the OpenSAFELY and EAVE II platforms subject to appropriate agreement, approvals and training as detailed in platform websites. Summary-level source data are provided with this paper.

Research involving human participants, their data, or biological material

Policy information about studies with human participants or human data. See also policy information about sex, gender (identity/presentation), and sexual orientation and race, ethnicity and racism.

Reporting on sex and gender	To mimic the randomisation of the target trial, we assumed randomisation conditional on several baseline covariates including sex. We used sex as biological attribute, and as reported in the electronic health records. We conducted subgroup analyses by sex (female versus male).
Reporting on race, ethnicity, or other socially relevant groupings	To mimic the randomisation of the target trial, we assumed randomisation conditional on several baseline covariates including ethnicity. Ethnicity was extracted through SNOMED CT codes and supplemented with information from secondary care records, and grouped into five broad categories (White, Black or Black British, Asian or Asian British, Mixed, Other) as recommended by latest electronic health records research in the UK. We conducted subgroup analyses by ethnicity.
Population characteristics	We provide extensive baseline characteristics, including sociodemographic and socioeconomic characteristics, see table 1 and table 2.
Recruitment	NA: Retrospective cohort of electronic health records
Ethics oversight	This study was approved by the Health Research Authority (REC reference 20/LO/0651) and by the LSHTM Ethics Board (reference 21863) and the National Research Ethics Service Committee, Southeast Scotland 02 (reference number: 12/SS/0201), and the Public Benefit and Privacy Panel for Health and Social Care (reference number: 1920-0279).

Note that full information on the approval of the study protocol must also be provided in the manuscript.

Field-specific reporting

Please select the one below that is the best fit for your research. If you are not sure, read the appropriate sections before making your selection.

☒ Life sciences ☐ Behavioural & social sciences ☐ Ecological, evolutionary & environmental sciences

For a reference copy of the document with all sections, see nature.com/documents/nr-reporting-summary-flat.pdf

Life sciences study design

All studies must disclose on these points even when the disclosure is negative.

Sample size	No sample size calculation was conducted - the target sample size was based on the maximum available data. We did not conduct any hypothesis testing and p-value reporting in the main analysis - only point estimates and their uncertainty. The sample size in this study is much large than previous clinical trials on this topic.
Data exclusions	The study population included adults (≥ 18 years old) who were hospitalised due to COVID-19 and prescribed either tocilizumab or sarilumab between July 1, 2021 and February 28, 2022. In July 2021, these two IL-6 inhibitors started being frequently prescribed for COVID-19 treatment and omicron peaked in January/February 2022. In OpenSAFELY, COVID-related admission was directly recorded in the COVID-19 therapeutics dataset, while in EAVE II admissions due to COVID-19 were identified as those occurring within 28 days of a positive Reverse Transcription Polymerase Chain Reaction (RT-PCR) test or those with an International Classification of Diseases, 10th Revision (ICD-10) code for COVID-19 (U07.1 and U07.2) in their admission record (in SMR01 and/or RAPID) as defined in previous studies
Replication	Code for data management and analysis in OpenSAFELY, the codelists and the pre-specified protocol, are archived online (https://github.com/opensafely/tocilizumab_and_sarilumab/tree/main) and all public logs of analysis activity are available online (https://jobs.opensafely.org/effectiveness-and-safety-of-covid-19-treatments-for-hospitalised-patients/tocilizumab_sarilumab/releases/).
Randomization	To mimic the randomisation of the target trial, we assumed randomisation conditional on the following baseline covariates: age (restricted cubic splines), sex (biological attribute, as reported in the electronic health records), NHS region, calendar time (restricted cubic splines), ethnicity (grouped into five broad categories: White, Black or Black British, Asian or Asian British, Mixed, Other), (Scottish) Index of Multiple Deprivation ([(S)IMD], as quintiles derived from the patient's postcode at lower super output area level), COVID-19 vaccination status (unvaccinated, one vaccination, two vaccinations, or three or more), SARS-CoV-2 re-infection status (positive test or clinical diagnosis code or exposure to COVID-19 drug at least three months prior), BMI, most recent record, grouped into <25.0, 25.0-<30.0, 30.0-<35, and >=35.0 kg/m2), previous use of other COVID-19 treatments (remdesivir, casirivimab/imdevimab and sotrovimab), diabetes, hypertension, chronic heart diseases, chronic respiratory diseases, moderate/severe renal disease, severe liver disease, solid cancer, hematological disease, immunosuppressive disease or treatment, and solid organ transplant. These covariates of interest, potentially prognostic for treatment initiation and outcome, were identified through literature review and discussions with domain experts. Comorbidities were identified through SNOMED CT codes in primary care records and ICD-10 in secondary care records. Ethnicity was identified through SNOMED CT codes and supplemented with information from secondary care records. Individuals with missing BMI, ethnicity, and (S)IMD were included with a missing indicator to maximise power, but alternative assumptions were tested in sensitivity analyses. Absence of recorded codes in terms of comorbidities, vaccination, reinfection, and prior COVID-19 treatment was assumed as not having such an event.
Blinding	NA: retrospective cohort study

Reporting for specific materials, systems and methods

We require information from authors about some types of materials, experimental systems and methods used in many studies. Here, indicate whether each material, system or method listed is relevant to your study. If you are not sure if a list item applies to your research, read the appropriate section before selecting a response.

Materials & experimental systems

n/a	Involved in the study	
☒	☐	Antibodies
☒	☐	Eukaryotic cell lines
☒	☐	Palaeontology and archaeology
☒	☐	Animals and other organisms
☐	☒	Clinical data
☒	☐	Dual use research of concern
☒	☐	Plants

Methods

n/a	Involved in the study	
☒	☐	ChIP-seq
☒	☐	Flow cytometry
☒	☐	MRI-based neuroimaging

Clinical data

Policy information about clinical studies

All manuscripts should comply with the ICMJE guidelines for publication of clinical research and a completed CONSORT checklist must be included with all submissions.

Clinical trial registration	NA.
Study protocol	The pre-specified protocol was archived online (https://github.com/opensafely/tocilizumab_and_sarilumab/tree/main).
Data collection	This study was based on a retrospective cohort of routinely collected electronic health records in England and Scotland; no active data collection was performed. The study population included adults (≥ 18 years old) who were hospitalised due to COVID-19 and prescribed either tocilizumab or sarilumab between July 1, 2021 and February 28, 2022.
Outcomes	The primary outcome was all-cause mortality within 28 days after treatment initiation, extracted from the ONS mortality database in OpenSAFELY and from the NRS mortality database in EAVE II. Secondary outcomes included 90-day all-cause mortality and time to

hospital discharge since treatment initiation. The date of tocilizumab or sarilumab prescription was defined as the baseline date. People were then followed from their baseline date until the earliest of either reaching the outcome, death (when analysing time to hospital discharge), or end of the follow-up period.

Plants

Seed stocks

NA

Novel plant genotypes

NA

Authentication

NA
