# Supplementary material for: Tocilizumab versus sarilumab among adults hospitalised with COVID-19: target trial emulation across England and Scotland
Source: Nat Commun. 2026 May 15;17:6453. doi: 10.1038/s41467-026-73134-9 (PMC13376390; doi:10.1038/s41467-026-73134-9)
Supplement: Supplementary file 3 — Transparent Peer Review file [file 41467_2026_73134_MOESM3_ESM.pdf]

Peer Review File

Tocilizumab versus sarilumab among adults hospitalised with COVID-19: target trial emulation across England and Scotland

Corresponding Author: Professor Laurie Tomlinson

This file contains all reviewer reports in order by version, followed by all author rebuttals in order by version.

Version 0:

Reviewer comments:

Reviewer #1

(Remarks to the Author)
The study by Zheng et al. using data from OpenSAFELY-TPP and EAVE II to evaluate differences between tocilizumab and sarilumab in relation to the outcomes of death and hospital discharge. I have the following comments:
1.While the authors note that the study was conducted during a period when no evidence was available and state that their findings support the use of these therapies, there is no control group to assess whether either treatment was effective during the study period.
If including an untreated control group is not feasible, this limitation should be clearly acknowledged and discussed in the manuscript.

2. Please include a cohort flow figure that indicates the number of participants excluded at each criterion. Specifically, please report the number of participants who were eligible for the emulated trial (e.g., hospitalized and met the criteria for treatment) but did not receive either tocilizumab or sarilumab.

3. Please clarify whether any action was taken to ensure that participants in both treatment arms were eligible to receive the treatment (e.g., if there was off-label use, if the proportions are similar in the two arms) and that the treatments were administered specifically for COVID-19 infection indication, not just with COVID-19.

4. For a study focused on hospitalized patients, please clarify why only covariates measured before hospitalization were collected and adjusted for.
The patient's severity at admission, represented by admission diagnosis, vital signs and laboratory values at admission, has not been considered, although these factors are strongly associated with outcomes and may have influenced treatment allocation.

5. Please revise the target trial emulation table and the report of target trial emulation following the recently published guideline (DOI: 10.1001/jama.2025.13350).
Please Ensure that both the target trial protocol and its emulation are clearly and correctly specified. For example, criteria such as corticosteroid use, ALT/AST levels, and platelet count are mentioned in the trial protocol but not reflected in the emulation. The assumption that all observed treatments followed the treatment guidelines outlined in the protocol is unlikely to always be correct. This highlights a potential lack of information necessary to fully emulate the specified trial.

6. Please include survival curves for the main outcomes examined.
In addition, please justify the use of hazard ratios in the analysis of acute outcomes and evaluate the proportional hazards assumption.

(Remarks on code availability)

Reviewer #2

(Remarks to the Author)
Key results
The manuscript presents a well-written target-trial-emulation comparing two treatments for COVID-19 using two national platforms (England and Scotland). It aims to infer comparative effectiveness of the treatments by emulating a randomized design in an observational setting.

Validity
• Since the authors frame the work as a target trial emulation, I recommend explicit adherence to the recent TARGET guideline for reporting (BMJ 2025; 390 doi: https://doi.org/10.1136/bmj-2025-087179 (Published 03 September 2025)). In particular, the manuscript would benefit from a table that contrasts the ideal randomized target trial (for example, the REMAP CAP study, Reference 8) with the current observational emulation across all relevant design elements (eligibility criteria, treatment strategies, assignment, follow-up, outcomes, causal contrasts, analysis).
• On lines 195–196 the statement "…providing real-world evidence to support clinical equivalence of both treatments" must be revised: the study was not explicitly designed as a formal equivalence study (no pre-specified equivalence margin). The authors should instead phrase the result as "consistent with comparable effectiveness" or similar.
• A potentially important covariate appears to be missing: days from symptom onset to treatment initiation. This variable may influence treatment decision and outcome. If it is not available in either platform, the authors should clarify why, and discuss the implications of its absence (e.g., residual confounding, selection bias).
• There is a substantial difference in COVID-19 mortality observed between the English and Scottish cohorts. The authors should provide plausible explanations (e.g., case-mix differences, healthcare system differences, data-coverage differences). For instance, the English platform covers about 40 % of the population (via OpenSAFELY) versus ~99 % in Scotland (via EAVE II). These discrepancies could reflect different representativeness and may impact generalizability.
• Please confirm and report whether there were any protocol deviations from the pre-specified emulation plan; if yes, how they were managed.

Significance
The work has strong potential significance: using large national-platform data to emulate a randomized trial is certainly relevant and timely for the COVID-19 therapeutic evidence base. However, to maximise its impact the authors should articulate clearly how their emulation adds beyond existing observational or trial evidence, and how well it meets the target-trial emulation framework.

Data and methodology
• The authors provide good description of data sources and analytic methods, but more detail is needed on key design decisions: e.g., how they defined treatment assignment, consequences of missing key timing variables (symptom onset), and how heterogeneity between platforms was addressed.

Clarity and context
• Overall the text is clear and accessible. The authors should consider re-structuring to emphasise upfront the target trial they are emulating (eligibility, interventions, comparators, outcome, follow-up) and how their observational design corresponds.
• It would strengthen the manuscript to situate the study explicitly in the context of previous work (both randomized trials and observational emulations), discussing limitations of observational emulations, and how they addressed them.
• References appear adequate, but the authors should verify whether key recent methodological literature on target-trial emulation reporting (including the TARGET guideline) is cited and discussed.

Suggested improvements
• Add a table comparing the hypothetical randomized target trial and the observational emulation, following the TARGET guideline.
• Rephrase the equivalence claim to avoid implying a formal equivalence design.
• Clarify availability of "days from symptom onset" and discuss its absence (or incorporate it if feasible).
• Provide discussion of the inter-platform mortality differences, including data-coverage and representativeness issues.
• Disclose any deviations from the protocol/emulation plan.
• Enhance transparency by describing sensitivity analyses and providing supplementary materials (covariate definitions, code availability) where possible.

My expertise
I have focussed on the statistical design, emulation of randomized trials, and observational causal inference. I did not evaluate the raw data themselves or full underlying code. Therefore, I may not have assessed all aspects of data integrity or low-level programming details.

(Remarks on code availability)

Version 1:

Reviewer comments:

Reviewer #1

(Remarks to the Author)
The authors have provided important edits to address my comments. Although some previously raised concerns have not be completely resolved because of data limitations, the authors have provided sufficient explanations and have appropriately acknowledged these limitations in the manuscript.
I have no further comments.

(Remarks on code availability)

Reviewer #2

(Remarks to the Author)
All of my remarks have been satisfactorily resolved, and the manuscript as a whole has been enhanced.

(Remarks on code availability)
I used an AI tool to review and check the code at https://github.com/opensafely/tocilizumab_and_sarilumab.

In the model part, PS weighting implementation uses raw 1/pscore weights (watch instability) with no stabilization (though later you do trimming in some sensitivity analyses). I recommend to use stabilized weights.

There are some minor potential bugs such as: A suspicious “previous_drug” derivation in Stata. This line is very likely not what was intended: gen previous_drug=(previous_drug_date=40)+(age>=60)

I recommend to check the code again.

Open Access This Peer Review File is licensed under a Creative Commons Attribution 4.0 International License, which permits use, sharing, adaptation, distribution and reproduction in any medium or format, as long as you give appropriate credit to the original author(s) and the source, provide a link to the Creative Commons license, and indicate if changes were made.
In cases where reviewers are anonymous, credit should be given to 'Anonymous Referee' and the source.
The images or other third party material in this Peer Review File are included in the article’s Creative Commons license, unless indicated otherwise in a credit line to the material. If material is not included in the article’s Creative Commons license and your intended use is not permitted by statutory regulation or exceeds the permitted use, you will need to obtain permission directly from the copyright holder.
To view a copy of this license, visit https://creativecommons.org/licenses/by/4.0/

Response to Reviewers

Tocilizumab versus sarilumab among adults hospitalised with COVID-19: target trial emulation across England and Scotland

We would like to thank all reviewers and the editor for their thorough assessment of our manuscript and their helpful comments, which we have very carefully considered. Please find our detailed point-by-point response below.

Reviewer #1

The study by Zheng et al. using data from OpenSAFELY-TPP and EAVE II to evaluate differences between tocilizumab and sarilumab in relation to the outcomes of death and hospital discharge. I have the following comments:

1. While the authors note that the study was conducted during a period when no evidence was available and state that their findings support the use of these therapies, there is no control group to assess whether either treatment was effective during the study period.
   If including an untreated control group is not feasible, this limitation should be clearly acknowledged and discussed in the manuscript.

   Response: *We would like to thank the reviewer for raising this point. We carefully searched again for evidence of the effectiveness of IL-6 inhibitors in the delta/omicron period. There has been a more recent trial, that recruited participants until June 2022 – providing some (however, uncertain) evidence. We have revised the Introduction to clarify this point (page 3, second paragraph).*
   However, the reviewer is right, we intentionally did not conduct an analysis comparing to an untreated control group due to methodological concerns. While our linked dataset includes rich longitudinal information with good national representativeness, the hospital episode records are limited in granularity. As a result, we are unable to fully ascertain all eligibility criteria for every hospitalized patient and therefore cannot reliably determine the total number of individuals who were eligible for the emulated trial but did not receive either tocilizumab or sarilumab. When comparing against a no-treatment group, as per target trial emulation guidance, establishing time zero among the untreated group is key to avoid design-related biases. Since this was not possible in our database, we refrained from such an analysis and focused on comparing two active treatments with clinical equipoise, and focus on this area as the main research gap.
   We acknowledge that this is an inherent limitation of our database and also related to other reviewer points below. We acknowledge this now as a limitation in the discussion (page 5, third paragraph):

   > "Third, the linked hospital episode data records are limited in granularity. As a result, we were unable to fully ascertain all eligibility criteria (e.g. platelet count, hypersensitivity to IL-6 or respiratory support). However, COVID-19 treatment with IL-6 inhibitors was strictly monitored and guided by clinical guidelines in the UK and, therefore, we assumed that receiving an IL-6 indicated no contraindication to these. More importantly, for a comparative effectiveness study evaluating two active treatments with identical clinical indications, both of which were available to clinicians during the study period, and used under prevailing clinical equipoise, the absence of such eligibility information is unlikely to introduce systematic bias."

2. Please include a cohort flow figure that indicates the number of participants excluded at each criterion. Specifically, please report the number of participants who were eligible for the emulated trial (e.g., hospitalized and met the criteria for treatment) but did not receive either tocilizumab or sarilumab.

   Response: *As mentioned above under point #1, the database limitation regarding eligibility criteria prevents us from reliably determining the total number of individuals who were eligible for the emulated trial but did not receive either tocilizumab or sarilumab and thereby constructing the desired cohort flow figure.*
   To clarify: For a comparative effectiveness study evaluating two active treatments with identical clinical indications, both of which were available to clinicians during the study period and used under prevailing clinical equipoise, the absence of this eligibility information is unlikely to introduce systematic bias. Our data extraction started with identifying the patients who received tocilizumab or sarilumab during the study period (as closely monitored and recorded in the national Blueteq system in England and the HEPMA system in Scotland).
   We had previously explained this in the text (section "Study population" and appendix Table S1 "eligibility criteria" in bold) but highlight it now also as a limitation (page 5, third paragraph), see exact wording of the limitation in our response to #1 above.

3. Please clarify whether any action was taken to ensure that participants in both treatment arms were eligible to receive the treatment (e.g., if there was off-label use, if the proportions are similar in the two arms) and that the treatments were administered specifically for COVID-19 infection indication, not just with COVID-19.

   Response: *Our IL-6 treatment data are from curated datasets in England and Scotland, summarizing all therapeutics used to specifically treat COVID-19 and no other condition (https://docs.opensafely.org/data-sources/therapeutics/). We have now clarified this point in the revised Methods (page 8, section "Interventions of Interest"):*

   > "In OpenSAFELY, this was ascertained from the COVID-19 therapeutics dataset based on the national Blueteq system, while in EAVE II this was ascertained from HEPMA as defined in prior research. Both treatment datasets summarized therapeutics specifically to treat COVID-19 and no other condition."

4. For a study focused on hospitalized patients, please clarify why only covariates measured before hospitalization were collected and adjusted for.
   The patient's severity at admission, represented by admission diagnosis, vital signs and laboratory values at admission, has not been considered, although these factors are strongly associated with outcomes and may have influenced treatment allocation.

   Response: *Thank you for this excellent point. As noted in our responses to #1 and #2 above, our in-hospital data sources are limited in granularity, thus we are unfortunately unable to adjust for laboratory values or vital signs at admission or prior to treatment allocation. However, given the clinical equipoise between the two IL-6 inhibitors and the shared eligibility criteria - particularly regarding disease severity - we believe that patients' medical history is likely more informative than additional baseline laboratory*

values. This was supported by the largely balanced baseline covariate between groups (Table 1) and the minimal change of effect estimates after adjusting for patients' demographic information, detailed medical history and COVID-19 related conditions (e.g., vaccination, re-infection, time between infection/testing positive and treatment initiation; Figure 2).
Nevertheless, we now explicitly acknowledge this as a limitation (page 5, third paragraph); please see our response to #1 above for the exact wording.

5. Please revise the target trial emulation table and the report of target trial emulation following the recently published guideline (DOI: 10.1001/jama.2025.13350).
   Please Ensure that both the target trial protocol and its emulation are clearly and correctly specified. For example, criteria such as corticosteroid use, ALT/AST levels, and platelet count are mentioned in the trial protocol but not reflected in the emulation. The assumption that all observed treatments followed the treatment guidelines outlined in the protocol is unlikely to always be correct. This highlights a potential lack of information necessary to fully emulate the specified trial.

   Response: *Thank you for drawing our attention to the recently published TARGET guideline. We had already included a similar table summarizing the target trial and its emulation (Appendix Table S1). However, we have now revised this table to follow the TARGET template (downloaded from* https://target-guideline.org/#resources*) and have replaced the RECORD-PE reporting guideline with the TARGET reporting guideline. We explained each target trial emulation element, including the eligibility criteria in detail. As mentioned under point #1 and #2, we have limited information with regards to our eligibility criteria – which we have added as a limitation; see exact wording of the limitation in our response to #1 above.*

6. Please include survival curves for the main outcomes examined.
   In addition, please justify the use of hazard ratios in the analysis of acute outcomes and evaluate the proportional hazards assumption.

   Response: *We agree with the reviewer and are aware of the limitation of hazard ratios. In our scenario, using hazard ratios to test a hypothesis at very specific timepoints is a suitable approach and commonly used in comparable randomized trials assessing time to death/discharge (e.g. Salama C, Han J, Yau L, et al. Tocilizumab in Patients Hospitalized with Covid-19 Pneumonia. N Engl J Med. 2021;384(1):20-30. doi:10.1056/NEJMoa2030340.)*
   Following the reviewer's suggestions, we have added the survival curves and the Schoenfeld residual plots (demonstrating proportional hazards) to the appendix (Figures S1 and S2) and referenced these figures in the Results chapter of the manuscript.

Reviewer #2

Key results

The manuscript presents a well-written target-trial-emulation comparing two treatments for

COVID-19 using two national platforms (England and Scotland). It aims to infer comparative effectiveness of the treatments by emulating a randomized design in an observational setting.

Validity

1. Since the authors frame the work as a target trial emulation, I recommend explicit adherence to the recent TARGET guideline for reporting (BMJ 2025; 390 doi: https://doi.org/10.1136/bmj-2025-087179 (Published 03 September 2025)). In particular, the manuscript would benefit from a table that contrasts the ideal randomized target trial (for example, the REMAP CAP study, Reference 8) with the current observational emulation across all relevant design elements (eligibility criteria, treatment strategies, assignment, follow-up, outcomes, causal contrasts, analysis).

   Response: *Thank you for pointing us towards the recently published TARGET guideline. We replaced the RECORD-PE reporting guideline with the TARGET reporting guideline. Moreover, we have adapted our preexisting appendix table S1 – which was already an attempt to report the target trial emulation elements – to exactly mirror the TARGET reporting template, downloaded from* https://target-guideline.org/#resources

2. On lines 195–196 the statement "…providing real-world evidence to support clinical equivalence of both treatments" must be revised: the study was not explicitly designed as a formal equivalence study (no pre-specified equivalence margin). The authors should instead phrase the result as "consistent with comparable effectiveness" or similar.

   Response: *We have revised the text as suggested, now stating (Conclusion, page 6):*

   > "In conclusion, among adults hospitalised due to COVID-19, we observed no difference between sarilumab versus tocilizumab with regards to death and hospital discharge, providing real-world evidence for comparable effectiveness of these two treatments."

3. A potentially important covariate appears to be missing: days from symptom onset to treatment initiation. This variable may influence treatment decision and outcome. If it is not available in either platform, the authors should clarify why, and discuss the implications of its absence (e.g., residual confounding, selection bias).

   Response: *Unfortunately, days from symptom onset is not available in our data platforms. We note that time since symptom onset was not an indication criterion when choosing between the two IL-6 inhibitors that were available in routine care at the time. Moreover, we had data on two relevant measures: "days since hospital admission" and "days since positive test", which were short (median 1 day) and comparable across groups. This is now acknowledged as a potential limitation in the revised Discussion (third paragraph, page 5)*

   > "Fourth, days from symptom onset to treatment initiation was not available. However, it is unlikely that days since symptom onset would have influenced the indication for one IL-6 inhibitor over the other, and both days since positive test

> and days since hospital admission were short (median of 1 day) and comparable between the two groups."

4. There is a substantial difference in COVID-19 mortality observed between the English and Scottish cohorts. The authors should provide plausible explanations (e.g., case-mix differences, healthcare system differences, data-coverage differences). For instance, the English platform covers about 40 % of the population (via OpenSAFELY) versus ~99 % in Scotland (via EAVE II). These discrepancies could reflect different representativeness and may impact generalizability.

   Response: *The higher mortality observed in the Scottish cohorts when compared to the English cohorts is most likely a reflection of case-mix differences. In particular, there are higher rates of obesity in Scotland than in England as has previously been reported (https://www.gov.scot/publications/scottish-health-survey-topic-report-uk-comparisons/). We added a paragraph in the discussion about this observation:*

   > "The higher mortality observed in the Scottish cohorts when compared to the English cohorts is most likely a reflection of case-mix differences. In particular, higher rates of obesity have consistently been observed across Scotland than in England - a risk factor severe COVID-19"

   Regarding the representativeness, we have further clarified in the Methods section (page 8) that, although OpenSAFELY-TPP covers only about 40% of the English population, previous work has highlighted the high representativeness of OpenSAFELY-TPP to the general population (e.g. compared against estimates from the Office of National Statistics): Andrews C, Schultze A, Curtis H, et al. OpenSAFELY: Representativeness of electronic health record platform OpenSAFELY-TPP data compared to the population of England. Wellcome Open Res. 2022;7:191. Published 2022 Jul 18. doi:10.12688/wellcomeopenres.18010.1

5. Please confirm and report whether there were any protocol deviations from the pre-specified emulation plan; if yes, how they were managed.

   Response: *Our analysis plan is part of the publicly available study protocol on GitHub (https://github.com/opensafely/tocilizumab_and_sarilumab/tree/main/docs). We have highlighted one important deviation in the sensitivity analysis regarding the propensity-score weighted Cox model. Due to the imbalance in one covariate (region), we decided to additionally adjust that model for the imbalanced covariate (in addition to the propensity score) and trimmed the propensity score at the lower 5th and upper 95th percentile. This post-hoc decision has already been described in the Results (see last paragraph, page 4).*

   There was one additional protocol deviation, which was a planned Bayesian Cox regression to inform the strength of evidence as a sensitivity analysis. We however dropped this analysis due to non-convergence of the model. We have now added this additional protocol deviation (see section "Statistical analysis", last sentence second paragraph page 9):

   > "As per protocol, we had planned to conduct a Bayesian Cox regression as additional sensitivity analysis to inform the evidence in favour or against the null

hypothesis. However, due to failed convergence of the model we had to drop this analysis."

Significance

6. The work has strong potential significance: using large national-platform data to emulate a randomized trial is certainly relevant and timely for the COVID-19 therapeutic evidence base. However, to maximise its impact the authors should articulate clearly how their emulation adds beyond existing observational or trial evidence, and how well it meets the target-trial emulation framework.

   Response: *No randomized clinical trial compared sarilumab versus tocilizumab during the delta/omicron period. REMAP-CAP compared the two IL-6 inhibitors, but the last recruited participant happened in April 2021. Our study extends this evidence base by covering a study period after the emergence of the delta and omicron SARS-CoV-2 variants and including largely vaccinated populations. We are not aware of any similar large-scale observational comparative effectiveness study of the two IL-6 inhibitors during the delta/omicron period. We have summarized the rationale for our study in the last paragraph of the Introduction and discuss the impact in the fourth paragraph of the Discussion (page 6).*
   We replaced the RECORD-PE reporting guideline with the TARGET reporting guideline. Moreover, we have adapted our preexisting appendix table S1 – which was an attempt to report the target trial emulation elements – to now exactly mirror the TARGET reporting template, downloaded from https://target-guideline.org/#resources

Data and methodology

7. The authors provide good description of data sources and analytic methods, but more detail is needed on key design decisions: e.g., how they defined treatment assignment, consequences of missing key timing variables (symptom onset), and how heterogeneity between platforms was addressed.

   Response: *We have added more details to the appendix table S1, which outlines all elements of the target trial emulation (and differences between the two platforms). We refrained from pooling the estimates across the two platforms due to heterogeneity considerations.*

Clarity and context

8. Overall the text is clear and accessible. The authors should consider re-structuring to emphasise upfront the target trial they are emulating (eligibility, interventions, comparators, outcome, follow-up) and how their observational design corresponds.

   Response: *We have now more explicitly provided all design details in the revised appendix table S1 to exactly mirror the TARGET template and hope that by replacing RECORD-PE with TARGET reporting guideline will increase clarity further.*

9. It would strengthen the manuscript to situate the study explicitly in the context of previous work (both randomized trials and observational emulations), discussing limitations of observational emulations, and how they addressed them.

Response: *We are not aware of any large-scale comparative effectiveness study of the two IL-6 inhibitors on COVID-19 outcomes – or any randomized trial during the delta/omicron period. The best evidence we are aware of is the network meta-analyses of randomized trials using direct and indirect evidence to infer comparative effectiveness (Zeraatkar D, Cusano E, Martínez JPD, et al. Use of tocilizumab and sarilumab alone or in combination with corticosteroids for covid-19: systematic review and network meta-analysis. BMJ Med. 2022 Feb 28;1(1):e000036. https://doi.org/10.1136/bmjmed-2021-000036 & Godolphin PJ, Fisher DJ, Berry LR, et al. Association between tocilizumab, sarilumab and all-cause mortality at 28 days in hospitalised patients with COVID-19: A network meta-analysis. PLOS ONE. 2022 Jul 8;17(7):e0270668. https://doi.org/10.1371/journal.pone.0270668) and the recent REMAP-CAP randomized trial (Derde L, Gordon AC, Mouncey PR, et al. Tocilizumab, sarilumab and anakinra in critically ill patients with COVID-19: a randomised, controlled, open-label, adaptive platform trial. Thorax. 2025 Aug 1;80(8):530–9. 10.1136/thorax-2024-222488). This evidence base is discussed in detail in the Discussion.*

10. References appear adequate, but the authors should verify whether key recent methodological literature on target-trial emulation reporting (including the TARGET guideline) is cited and discussed.

    Response: *We have replaced the RESPOND-PE with the TARGET reporting guideline and referenced it accordingly, see reference 17.*

Suggested improvements

11. Add a table comparing the hypothetical randomized target trial and the observational emulation, following the TARGET guideline.

    Response: *We revised appendix table S1 to exactly mirror the TARGET template.*

12. Rephrase the equivalence claim to avoid implying a formal equivalence design.

    Response: *We rephrased the equivalence claim, please see our response to comment #2.*

13. Clarify availability of "days from symptom onset" and discuss its absence (or incorporate it if feasible).

    Response: *Please see our response to Comment #3 above.*

14. Provide discussion of the inter-platform mortality differences, including data-coverage and representativeness issues.

    Response: *Please see our response to Comment #4 above.*

15. Disclose any deviations from the protocol/emulation plan.

Response: *Please see our response to Comment #5 above.*

16. Enhance transparency by describing sensitivity analyses and providing supplementary materials (covariate definitions, code availability) where possible.

Response: *These details are now provided in the revised appendix table S1. Regarding code availability we refer to our section "Data availability and code availability".*

Response to Reviewers | Round #2

Tocilizumab versus sarilumab among adults hospitalised with COVID-19: target trial emulation across England and Scotland

Reviewer #1
The authors have provided important edits to address my comments. Although some previously raised concerns have not be completely resolved because of data limitations, the authors have provided sufficient explanations and have appropriately acknowledged these limitations in the manuscript. I have no further comments.

> ***Response:*** *Thank you very much!*

Reviewer #2
All of my remarks have been satisfactorily resolved, and the manuscript as a whole has been enhanced. I used an AI tool to review and check the code at https://github.com/opensafely/tocilizumab_and_sarilumab.

a) In the model part, PS weighting implementation uses raw 1/pscore weights (watch instability) with no stabilization (though later you do trimming in some sensitivity analyses). I recommend to use stabilized weights.

> ***Response:*** *We thank the reviewer for this recommendation. We would like to clarify that in the trade-off between better balancing in covariate distributions between groups (avoiding confounding by indication) and better variance stability, we chose to emphasize the former because that PS weighted analysis is a sensitivity analysis and we want to obtain an unbiased effect estimate. That also ensures that we do not deviate from our original analysis plan posted online. As the reviewer points out, we also did trimming in later analysis which partially addresses this point.*

b) There are some minor potential bugs such as: A suspicious "previous_drug" derivation in Stata. This line is very likely not what was intended: gen previous_drug=(previous_drug_date=40)+(age>=60)
I recommend to check the code again.

> ***Response:*** *We thank the reviewer for checking our codes and we re-checked the "previous_drug" coding that AI identified as a potential issue. However, actually there is not a line like that (gen previous_drug=(previous_drug_date=40)+(age>=60)) so we think it's likely an AI hallucination. In addition, our codes have undergone independent checks between two authors of this study.*
